# Biocompatible AIEgen/p-glycoprotein siRNA@reduction-sensitive paclitaxel polymeric prodrug nanoparticles for overcoming chemotherapy resistance in ovarian cancer

**DOI:** 10.7150/thno.53828

**Published:** 2021-01-27

**Authors:** Jun Wu, Quan Wang, Xiaoqi Dong, Min Xu, Juliang Yang, Xiaoqing Yi, Biao Chen, Xiyuan Dong, Ying Wang, Xiaoding Lou, Fan Xia, Shixuan Wang, Jun Dai

**Affiliations:** 1 Engineering Research Center of Nano-Geomaterials of the Ministry of Education, Faculty of Materials Science and Chemistry, China University of Geosciences, Wuhan 430074, China.; 2 Department of Obstetrics and Gynecology, Tongji Hospital, Tongji Medical College, Huazhong University of Science and Technology, Wuhan 430030, China.; 3 College of Pharmacy, Gannan Medical University, Ganzhou 341000, China.; 4 Department of Pathology, Tongji Hospital, Tongji Medical College, Huazhong University of Science and Technology, Wuhan 430030, China.

**Keywords:** chemotherapy resistance, ovarian cancer, p-glycoprotein, aggregation-induced emission, drug delivery

## Abstract

Nanoparticle drug delivery system (NDDS) is quite different from the widely studied traditional chemotherapy which suffers from drug resistance and side effect. NDDS offers the straightforward solution to the chemotherapy problem and provides an opportunity to monitor the drug delivery process in real time. In this vein, we developed one NDDS, namely Py-TPE/siRNA@PMP, to relieve resistance and side effects during chemotherapy against ovarian cancer. The Py-TPE/siRNA@PMP is a multifunctional polymeric nanoparticle contained several parts as follows: (1) a nanoparticle (NP) self-assembled by reduction-sensitive paclitaxel polymeric prodrug (PMP); (2) the glutathione (GSH)-responsive release of paclitaxel (PTX) for the suppression of ovarian cancer cells; (3) the P-glycoprotein (P-gp) siRNA for restoring the sensitivity of chemo-resistant tumor cells to chemotherapy; (4) the positively charged aggregation-induced emission fluorogen (AIEgen) Py-TPE for tumor imaging and promoting encapsulation of siRNA into the nanoparticle.

**Methods**: The Py-TPE/siRNA@PMP nanoparticles were prepared by self-assembly method and characterized by the UV-Vis absorption spectra, zeta potentials, TEM image, stability assay and hydrodynamic size distributions. The combinational therapeutic effects of Py-TPE/siRNA@PMP on overcoming chemotherapy resistance were explored both* in vitro* and* in vivo.*

**Result**: The Py-TPE/siRNA@PMP exhibited an average hydrodynamic size with a good stability. Meanwhile they gave rise to the remarkable chemotoxicity performances *in vitro* and suppressed the tumors growth in both SKOV-3/PTX (PTX resistance) subcutaneous and intraperitoneal metastasis tumor models. The investigations on ovarian cancer patient-derived xenografts (PDX) model revealed that Py-TPE/siRNA@PMP was able to effectively overcome their chemo-resistance with minimal side effects.

**Conclusion:** Our findings demonstrated the Py-TPE/siRNA@PMP as a promising agent for the highly efficient treatment of PTX-resistant cells and overcoming the shortage of chemotherapy in ovarian cancer.

## Introduction

Ovarian cancer is one of the prevalent cancers in gynecology with five-year survival rate below 50% and causes the highest mortality rate among gynecological tumors 1, 2. Most ovarian cancer is sensitive to first-line chemotherapy drug in the initial stage, such as paclitaxel (PTX) 3. However, chemotherapy drugs are not consistently effective because of quick drug-resistance in the development of tumors 4. On the other hand, uncontrolled tumors need second-line chemotherapy to elicit ever more side effect. Such failure of repeat chemotherapy elongates the patients' sufferings and narrows the clinical benefits. Therefore, the development of new reagents for overcoming drug resistance of chemotherapy is intriguing and urgently demanded.

Nanoparticle drug delivery system (NDDS) is widely used to reduce chemotherapy resistance and side effects. In recent ten years, a biocompatible amphiphilic polymer has been employed to construct a high-performance drug delivery system 5-7. Because of self-assembly ability, the amphiphilic polymer was able to aggregate into varieties of nanoparticles (NPs) and possess multiple advantages including high chemical stability and rich loading content 8,9. Moreover, the amphiphilic polymer-based NP function as a bioavailability vessel to improve enhanced permeability and retention (EPR) effect and selectively target tumor cells, which led to the decrease of side effects 10-13. In comparison to the traditional method, NDDS with polymeric biocomponents were more favorable for tumor treatment. The responsive polymeric prodrug NPs has been especially applied for releasing drugs under stimulation, endowing the drug delivery with desired time and space 14,15. For example, the intracellular concentration (2-10 ×10^-3^ M) of glutathione (GSH) in cancer cells is about a thousand-fold than that within extracellular tumor microenvironment (2-20 × 10^-6^ M) 16 and a stimuli responsive polymeric NPs could be fulfilled by sensing GSH. Herein, the GSH-sensitive polymeric prodrug NP was a potential controlled cargo carrier to achieve an effective restrain of side effects in normal tissues.

On the other hand, P-glycoprotein (P-gp) is an ATP-binding cassette transporter on the cell membrane that constantly searches for exogenous foreign molecules and pumps them out of the cells. The active efflux function of P-gp exhibited a key defense mechanism of drug resistance in ovarian cancer 17,18 and down-regulations of P-gp were proved to restore the sensitivity of tumor cells to chemotherapy. In this vein, mesoporous silica NPs loading P-gp siRNA and doxorubicin have been constructed for responsive breast cancer therapy and elimination of drug resistance problem *in vivo* 19. Patil' group developed PLGA-PEI NPs encapsulated with P-gp siRNA and PTX for an efficient treatment in drug-resistant tumor cells 20. A delivery of P-gp siRNA displayed excellent biomedical properties for relieving the drug resistance of tumors.

**Scheme 1:**
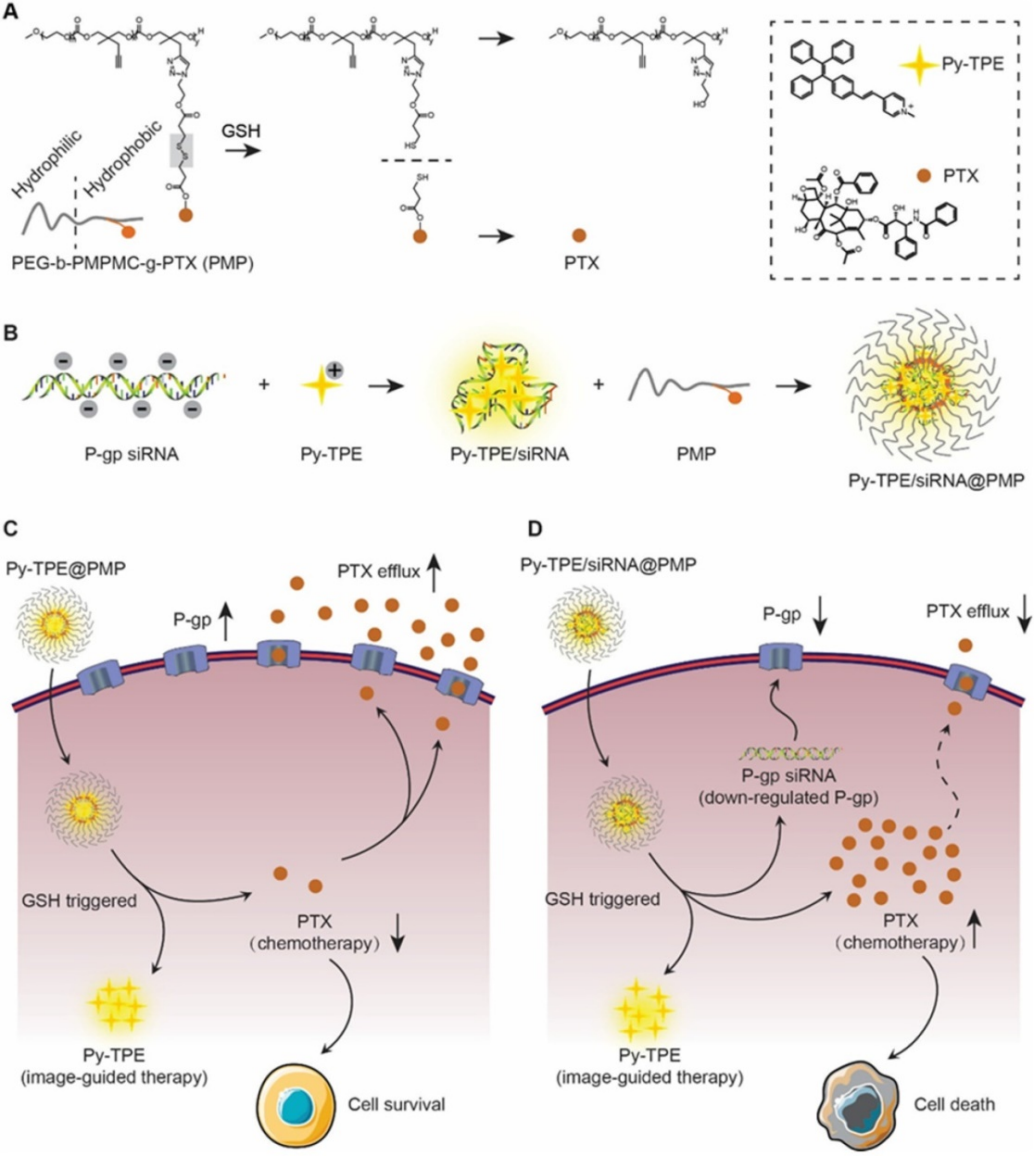
(A) PMP released PTX under the GSH stimulation. (B) P-gp siRNA and Py-TPE formed a Py-TPE/siRNA complex by electrostatic interaction, which was encapsulated by PMP to construct the Py-TPE/siRNA@PMP. (C) Py-TPE@PMP led to an unsuccessful treatment of SKOV-3 /PTX (PTX resistance) cells with high P-gp expression. (D) Py-TPE/siRNA@PMP down-regulated the P-gp expression of SKOV-3/PTX cells and reduced the efflux of PTX, finally overcoming the chemotherapy resistance of ovarian cancer.

Inspired by the strategy of responsive polymeric NPs 21,22, we constructed a reduction-sensitive NDDS, namely Py-TPE/siRNA@PMP. The amphiphilic polymer skeleton was developed based on poly (ethylene glycol)-b-poly(5-mthyl-5-propargyl-1,3-dioxan-2-one)-g-paclitaxel (PMP), which conjugated with PTX via disulfide linker to obtain a reduction-sensitive polymeric prodrug. Driven by electrostatic interactions, the negative charge of siRNA and positively charged Py-TPE were incorporated into Py-TPE/siRNA@PMP for image-guided gene delivery 23,24. As shown in Scheme 1, Py-TPE/siRNA@PMP NPs were prepared via self-assembly and able to afford long-lasting circulation in the blood stream. After being intravenously injected into PTX-resistant models or PDX models (ovarian cancer patient-derived xenografts), Py-TPE/siRNA@PMP was accumulated at tumor site by a passive manner of EPR effect. Then high concentration of GSH in tumor cells facilitated the cleavage of the disulfide bond and the subsequent release of active PTX, fluorescent agent Py-TPE and P-gp siRNA. As a result, PTX interacted with microtubule to prevent its normal function, and P-gp siRNA aimed to retard the transport of PTX out of cells, which together disrupted the cancer cell replication and eventually led to the apoptosis 25,26. The Py-TPE further enabled the discrimination between the cancer and normal cells by different fluorescent contrast, rendering an image-guided chemotherapy 27-32. By virtue of such a rational design, Py-TPE/siRNA@PMP was developed by altering the drug-resistance and side effects of chemotherapy 33,34 to provide a potential therapeutic option for ovarian cancer.

## Results and Discussion

### Synthesis and characterization of Py-TPE/siRNA@PMP

**Figure 1:**
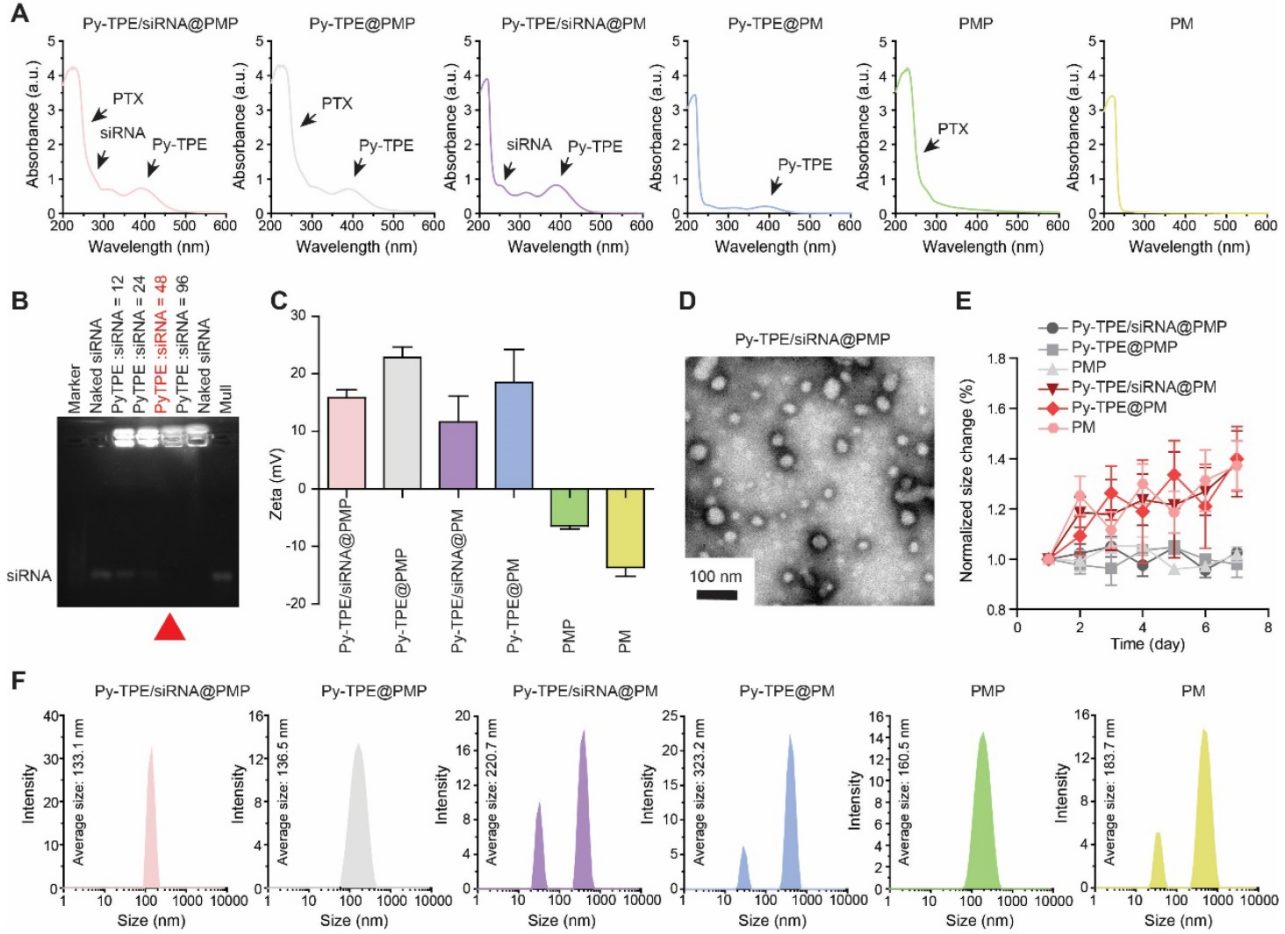
(A) UV-Vis absorption spectra of Py-TPE/siRNA@PMP, Py-TPE@PMP, Py-TPE/siRNA@PM, Py-TPE@PM, PMP and PM. (B) The optimum weight ratios (red arrow) of P-gp siRNA to Py-TPE in Py-TPE/siRNA@PMP were determined by agarose gel electrophoresis. (C) Zeta potentials of Py-TPE/siRNA@PMP, Py-TPE@PMP, Py-TPE/siRNA@PM, Py-TPE@PM, PMP and PM. (D) TEM image of Py-TPE/siRNA@PMP. Scale bar: 100 nm. (E) Stability assays of Py-TPE/siRNA@PMP, Py-TPE/siRNA@PM, Py-TPE@PMP, Py-TPE@PM, PMP and PM in PBS for 7 days at room temperature. (F) Hydrodynamic size distributions of Py-TPE/siRNA@PMP, Py-TPE@PMP, Py-TPE/siRNA@PM, Py-TPE@PM, PMP and PM.

PMP and Py-TPE were synthesized according to our reported procedures (Scheme S1) 35,36. ^1^H NMR spectra and mass spectrometry were confirmed in Figures S1-S4. UV-Vis (ultraviolet-visible) absorption bands of Py-TPE, P-gp siRNA and PTX were illustrated in Figure S5 with three characteristic absorption peaks at 393 nm, 260 nm and 256 nm, respectively. Py-TPE/siRNA@PMP displayed the triple absorption peaks at 393 nm, 260 nm and 256 nm, which were in according with the characteristic peaks of Py-TPE, P-gp siRNA and PTX, respectively, suggestive of a successful preparation (Figure 1A). Any NPs containing Py-TPE emitted the fluorescent wavelength at ~600 nm (Figure S6). Gel electrophoresis revealed an optimal weight ratio of 48 between Py-TPE and siRNA (Figure 1B), proving that P-gp siRNA had been successfully encapsulated into Py-TPE/siRNA@PMP 37. The drug loading capacity of three elements in Py-TPE/siRNA@PMP were also determined by UV-Vis spectrophotometer, and the concentrations of Py-TPE, P-gp siRNA and PTX were 222 μg/mL, 4.63 μg/mL and 382.3 μg/mL, respectively (Figure S7-8, Figure 1B). The zeta potentials of Py-TPE/siRNA@PMP, Py-TPE@PMP, Py-TPE/siRNA@PM, Py-TPE@PM, PMP and PM were 15.81 mV, 22.83 mV, 11.59 mV, 18.57 mV, -6.38 mV, -13.63 mV, respectively (Figure 1C). After loading with the P-gp siRNA, the zeta potential of Py-TPE/siRNA@PMP was much lower than that of Py-TPE@PMP 38. The results from the electrophoresis and zeta potential both proved that P-gp siRNA was able to interact with Py-TPE *via* the electrostatic interactions and turn into a stable complex encased by PMP.

In the transmission electron microscopy (TEM) image, Py-TPE/siRNA@PMP was uniform spherical-shaped NPs with a diameter of around 50 nm (Figure 1D). In dynamic light scattering (DLS), the Py-TPE/siRNA@PMP, Py-TPE@PMP and PMP kept the intact spherical morphology during 7 days at room temperature (Figure 1E). However, at the same condition, the hydrodynamic sizes of Py-TPE/siRNA@PM, Py-TPE@PM and PM varied dramatically (Figure 1E). The PEG chain of PM was functionalized to conjugate PTX, a hydrophobic drug, providing the ability to self-assemble in dense layer that excluded water due to the hydrophobic core and enhanced stability in aqueous solution. Meanwhile, in comparison to PM groups, PMP-based NPs were more homogeneous (Figure 1F), and Py-TPE/siRNA@PMP maintained a stable particle size whether in aqueous solution or FBS containing culture solution (Figure S9). Because of the shrinkage of PEG shells after drying, the size of Py-TPE/siRNA@PMP in TEM was smaller than that in DLS (Figure 1D and 1F) 22.

More experiments have been conducted to prove that PTX, siRNA, and Py-TPE could be release under the concentration of GSH in tumor cells but not under blood circulation. The GSH concentrations in tumor cells and in blood circulation were selected as 10 mM and 10 μM, respectively 37. After incubation with 10 mM GSH for 24 hours, PTX could be detected by mass spectroscopy (Figure S10). However, after 24 hours of incubation with 10 μM GSH, almost no PTX was detected, indicating that a release of PTX only occurred under the high concentration of GSH in tumor cells. Similarly, the release of Py-TPE and siRNA were evaluated under the same conditions (Figure S11 and S12), which suggested that Py-TPE and siRNA were only released under the high concentration of GSH in tumor cells but not under blood circulation.

### Py-TPE/siRNA@PMP overcame chemotherapy resistance of ovarian cancer *in vitro*

**Figure 2:**
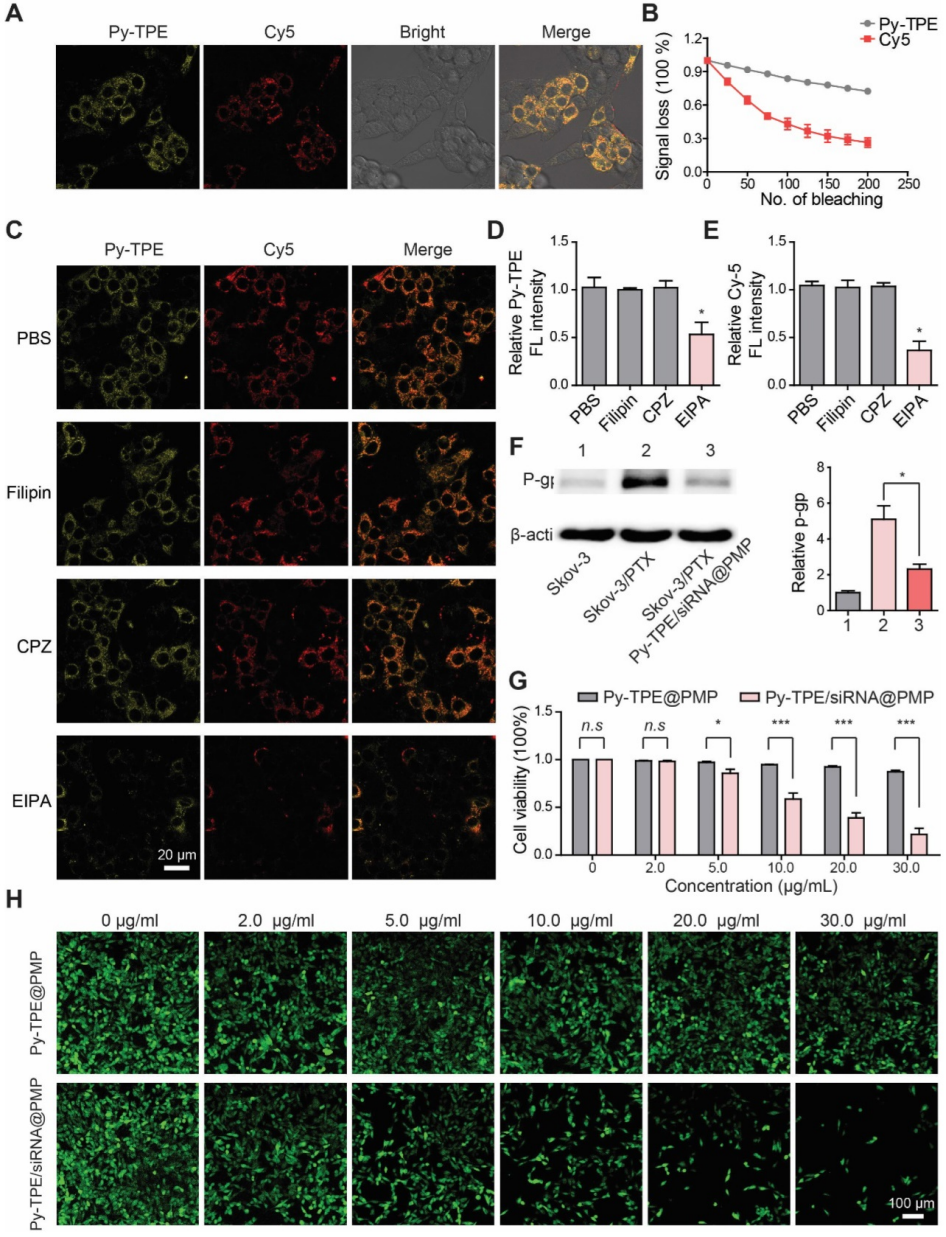
(A) After incubation of SKOV-3/PTX cells with Py-TPE/siRNA-Cy5@PMP, CLSM was used to detect the fluorescence localization of Py-TPE and Cy5 in SKOV-3/PTX cells. Py-TPE: Ex 405 nm, Em 600 nm. Cy5: Ex 633 nm, Em 680 nm. Scale bar: 10 nm. (B) Photostability of Py-TPE and Cy5 in living cells. Signal loss (%) of fluorescent emission of Py-TPE and Cy5 with the increasing number of bleaching. (C) PBS, Filipin, CPZ and EIPA were applied to test the cellular uptake efficiency of Py-TPE/siRNA-Cy5@PMP. The relative intensity of (D) Py-TPE and (E) Cy5 in SKOV-3/PTX cells. (F) The expressions of P-gp from SKOV-3, SKOV-3/PTX and SKOV-3/PTX cells were detected by western blot and analyzed quantitatively when treated with Py-TPE/siRNA@PMP. (G) The viabilities of SKOV-3/PTX cells were conducted by treating cells with Py-TPE@PMP or Py-TPE/siRNA@PMP. (H) CMFDA was introduced to detect the effect of different concentrations of Py-TPE@PMP or Py-TPE/siRNA@PMP on the viability of SKOV-3/PTX cells. CMFDA: Ex 488 nm, Em 540 nm. Data were reported as mean ± SD and analyzed by two-sided Student's t-test. * *p*< 0.05, *** *p*< 0.001, *n.s.* not significant.

First, in order to study the therapeutic responses of Py-TPE/siRNA@PMP to chemotherapy-resistance ovarian cancer cells, SKOV-3/PTX (PTX-resistant SKOV-3) cell lines were developed 39. In Figure S13, SKOV-3 was sensitive to PTX at the early stage of treatment. After 30-day culturing with PTX, the remaining SKOV-3 cells showed a complete drug resistance to PTX, indicating the successful establishment of SKOV-3/PTX cells. By using confocal laser scanning microscope (CLSM), the fluorescence intensities of Py-TPE/siRNA@PMP were closely correlated to the incubation time and initial NPs concentration (Figure S14 and S15). Second, siRNA was tagged with Cy5 to yield the Py-TPE/siRNA-Cy5@PMP, which consisted of two fluorescence agents (Cy5 and Py-TPE) for the specific imaging of cancer cells. Both of fluorescence signals of Py-TPE and Cy5 were observed in SKOV-3/TPX cells, indicating a successful delivery of P-gp siRNA into the cells (Figure 2A). The good photostability of AIEgens provided the long-term imaging ability of Py-TPE/siRNA-Cy5@PMP, allowing its better tracking performance than the Cy5 (Figure 2B).

To explore the mechanism of cellular uptake of Py-TPE/siRNA@PMP, SKOV-3/PTX cells were cultured with PBS (control), CPZ (clathrin-mediated endocytosis inhibitor), Filipin (caveolae-mediated endocytosis inhibitor) and EIPA (macropinocytosis inhibitor), respectively (Figure 2C). The SKOV-3/PTX cells exhibited strong fluorescence but faintly fluoresced only when treated with EIPA, which indicated that Py-TPE/siRNA-Cy5@PMP was delivered into SKOV-3/PTX cells with the help of macropinocytosis (Figure 2D/2E). A preferential prevention of PTX efflux in cell membrane was achieved by Py-TPE/siRNA@PMP (Figure 2F). At the same time, the cell viabilities of SKOV-3/PTX cells were significantly decreased with the increased concentrations of Py-TPE/siRNA@PMP but almost unchanged with the Py-TPE@PMP treatment, even with 30 μg/mL (Figure 2G), suggestive of a highly efficient therapy of Py-TPE/siRNA@PMP in SKOV-3/PTX cells.

SKOV-3/PTX cells were co-transfected with CMFDA (5-chloromethylfluorescein diacetate, a living cell tracer) to further illustrate the cell viability by treatment of Py-TPE/siRNA@PMP and Py-TPE@PMP. The fluorescent signal of living cells gradually vanished with the increase in concentration of Py-TPE/siRNA@PMP, while remained strong emission by using Py-TPE@PMP (Figure 2H). Moreover, PI (propidium iodide) and Annexin V-FITC staining assay were used to investigate the cell apoptosis by PMP, Py-TPE/siRNA@PM and Py-TPE/siRNA@PMP, respectively. As a result, when only treated with Py-TPE/siRNA@PMP, PI and Annexin V-FITC strongly fluoresced (Figure S16), while PMP and Py-TPE/siRNA@PM group exhibited faint fluorescence (Figure S16), suggesting that Py-TPE/siRNA@PMP, not PMP and Py-TPE/siRNA@PM, was able to cause cellular death. More importantly, even when treated with 30 μg/ml Py-TPE/siRNA@PMP NPs, 80% of normal cells (human lung fibroblast cells) survived, which may be related to low levels of GSH in normal cells (Figure S17).

Last but not least, the effects on microtubes in SKOV-3 cells and SKOV-3/PTX cells under the treatment of PBS, PTX, PMP, Py-TPE/siRNA@PM and Py-TPE/siRNA@PMP, respectively, were conducted in Figure S18. Compared to the control group (PBS), almost no obvious morphological changes of microtubules were detected when incubated with PBS and PM NPs. However, the peripheral microtubules were moderately smeared when cells were incubated with PTX, PMP and Py-TPE/siRNA@PMP for 8 h, which suggested that only PTX prevent the function of microtube in cells.

### Pharmacokinetics and bio-distribution of Py-TPE/siRNA@PMP *in vivo*

**Figure 3:**
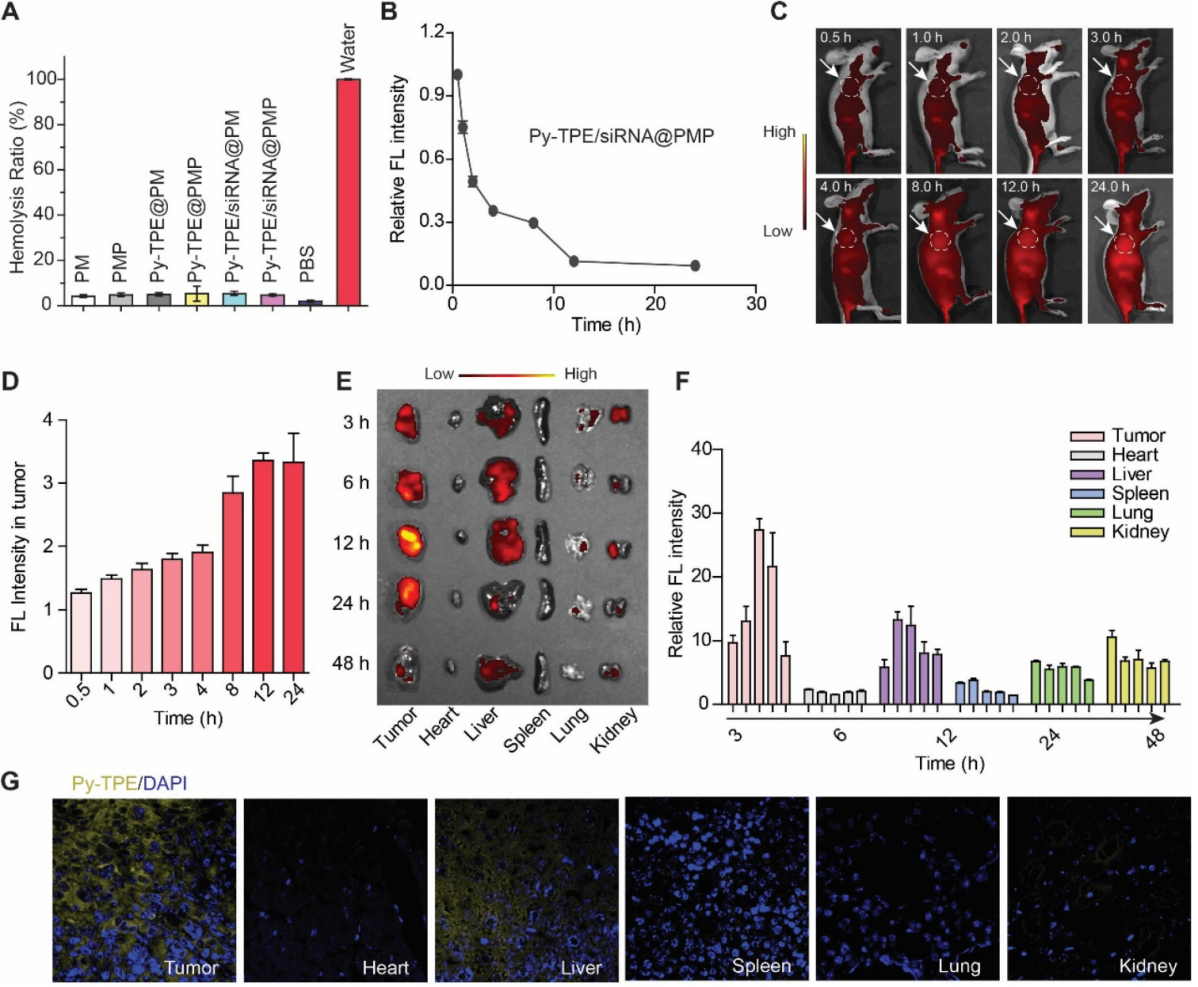
(A) Hemolysis test of PM, PMP, Py-TPE@PM, Py-TPE@PMP, Py-TPE/siRNA@PM, Py-TPE/siRNA@PMP, PBS and water. (B) The pharmacokinetics of Py-TPE/siRNA@PMP *in vivo*. (C) *In situ* real-time imaging of Py-TPE/siRNA@PMP NPs in SKOV-3/PTX cell tumor bearing mice. The area within the white dotted line was the location of the tumor. (D) The Py-TPE fluorescence intensity of tumor area was quantitatively analyzed. (E) The bio-distribution of Py-TPE/siRNA@PMP *in vivo*. Ex =430 nm, Em =600 nm. (F) Quantification of the bio-distribution of Py-TPE/siRNA@PMP *in vivo*. (G) The images of Py-TPE/siRNA@PMP in tumor or organ was detected by CLSM.

For the safety testing, the hemolysis detection of NPs in live animals are specially required 40,41. As shown in Figure 3A, there was almost no significant hemolysis occurred (less than 5% hemolysis rate) in all groups, which suggested their biocompatibility for intravenous injection in mice models. Second, the pharmacokinetics results revealed that up to 30% Py-TPE/siRNA@PMP was constantly circulating in the blood stream for 8 h after injection (Figure 3B). Such a long-term circulation was able to enhance EPR effect 42,43 and delay the elimination by mononuclear phagocyte system 44,45. Third, *in situ* real-time imaging were further performed in tumor bearing mice (Figure 3C), and the fluorescence intensity of Py-TPE/siRNA@PMP displayed a fluorescence maximum at tumor site at 12 h after injection (Figure 3D), indicating that excellent photostability of Py-TPE/siRNA@PMP for specific long-term tumor site imaging. Moreover, SKOV-3/PTX subcutaneous tumor models were generated to study the bio-distribution of Py-TPE/siRNA@PMP *in vivo* (Figure 3E). The most intense fluorescence signal of tumors was observed at 12 h and gradually vanished in 48 h. Except for the tumor site, the administrated NPs were enriched in liver and kidney (Figure 3F). Meanwhile, the micro-distribution of Py-TPE/siRNA@PMP in tumor and different organs were directly quantified by CLSM, and the aggregates of NPs almost distributed on tumors, followed by liver, but no signal detected in lung, kidney, spleen and heart (Figure 3G). In this vein, Py-TPE/siRNA@PMP demonstrated the remarkable pharmacokinetics and bio-distribution *in vivo*.

### Py-TPE/siRNA@PMP overcame chemotherapy resistance of ovarian cancer *in vivo*

**Figure 4:**
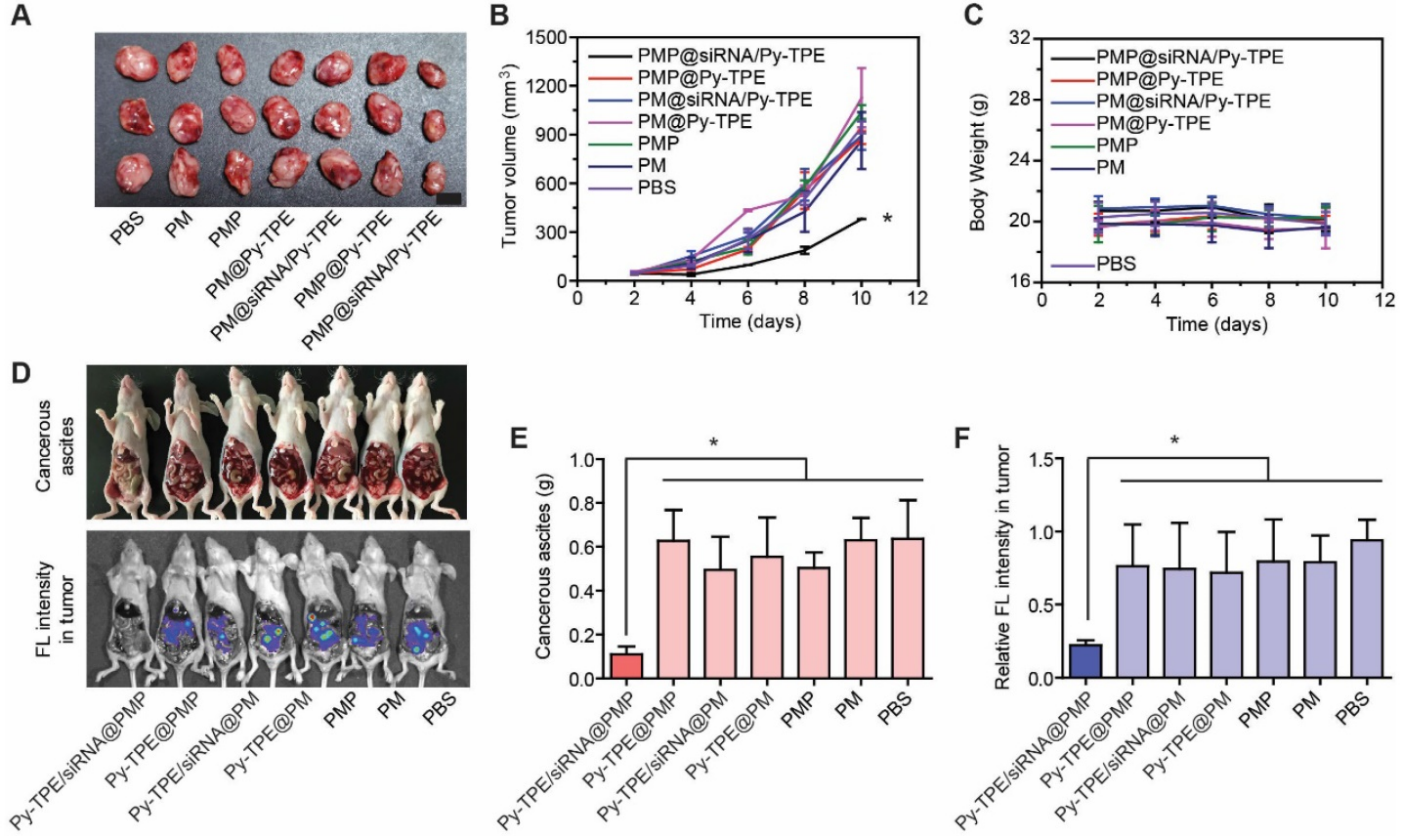
(A) SKOV-3/PTX mice tumors changed during treatment of PBS (control), PM, PMP, Py-TPE@PM, Py-TPE/siRNA@PM, Py-TPE@PMP and Py-TPE/siRNA@PMP, respectively. (B) Relative tumor volume of (A) during the therapy under 2-10 days. (C) Body weight of mouse during the treatment. (D) The blood ascites (Top) and metastasis (bottom) in SKOV-3/PTX intraperitoneal metastasis mice after treated with PM, PMP, Py-TPE@PM, Py-TPE@PMP, Py-TPE/siRNA@PM, Py-TPE/siRNA@PMP and PBS, respectively. (E/F) The quantitative analysis of blood ascites of (D, top) and metastasis (D, bottom). Data were reported as mean ± SD and analyzed by two-sided Student's t-test. * *p*< 0.05.

In order to investigate the therapeutic effect of Py-TPE/siRNA@PMP *in vivo*, we established three animal models, including the SKOV-3/PTX subcutaneous tumor model, SKOV-3/PTX intraperitoneal metastasis model and patient-derived xenografts (PDX) tumor model 39, 45. As shown in Figure 4A and 4B, in comparison of other groups, the growth of tumor in SKOV-3/PTX subcutaneous tumor models was suppressed by treating Py-TPE/siRNA@PMP. While Py-TPE@PMP, Py-TPE/siRNA@PM, Py-TPE@PM, PMP and PM groups were lowly active or inactive in restraint of the tumor growth (Figure 4A and 4B), suggestive of the effective inhibition of tumor by combinational treatment of PTX and P-gp siRNA. Because the bloody ascites is closely related to cancers' progression and metastasis 46, the investigation on abdominal cavities of mice for polymeric NPs were carried out at 14th day after intraperitoneal injection (Figure 4D, top). There was a large amount of blood ascites in Py-TPE@PMP (0.63 ± 0.14 g), Py-TPE/siRNA@PM (0.49 ± 0.15 g), Py-TPE@PM (0.55 ± 0.18 g), PMP (0.50 ± 0.07 g), PM (0.63 ± 0.10 g) and PBS (0.64 ± 0.18 g) groups, while there was almost no blood ascites in Py-TPE/siRNA@PMP (0.11 ± 0.03 g) group (Figure 4E). Similar responses were also given by suppression of intraperitoneal metastasis models in SKOV-3/PTX mice (Figure 4D, bottom), which reflected the significant role of Py-TPE/siRNA@PMP to inhibit intraperitoneal metastasis as well as blood ascites in ovarian cancer cells (Figure 4F).

**Figure 5:**
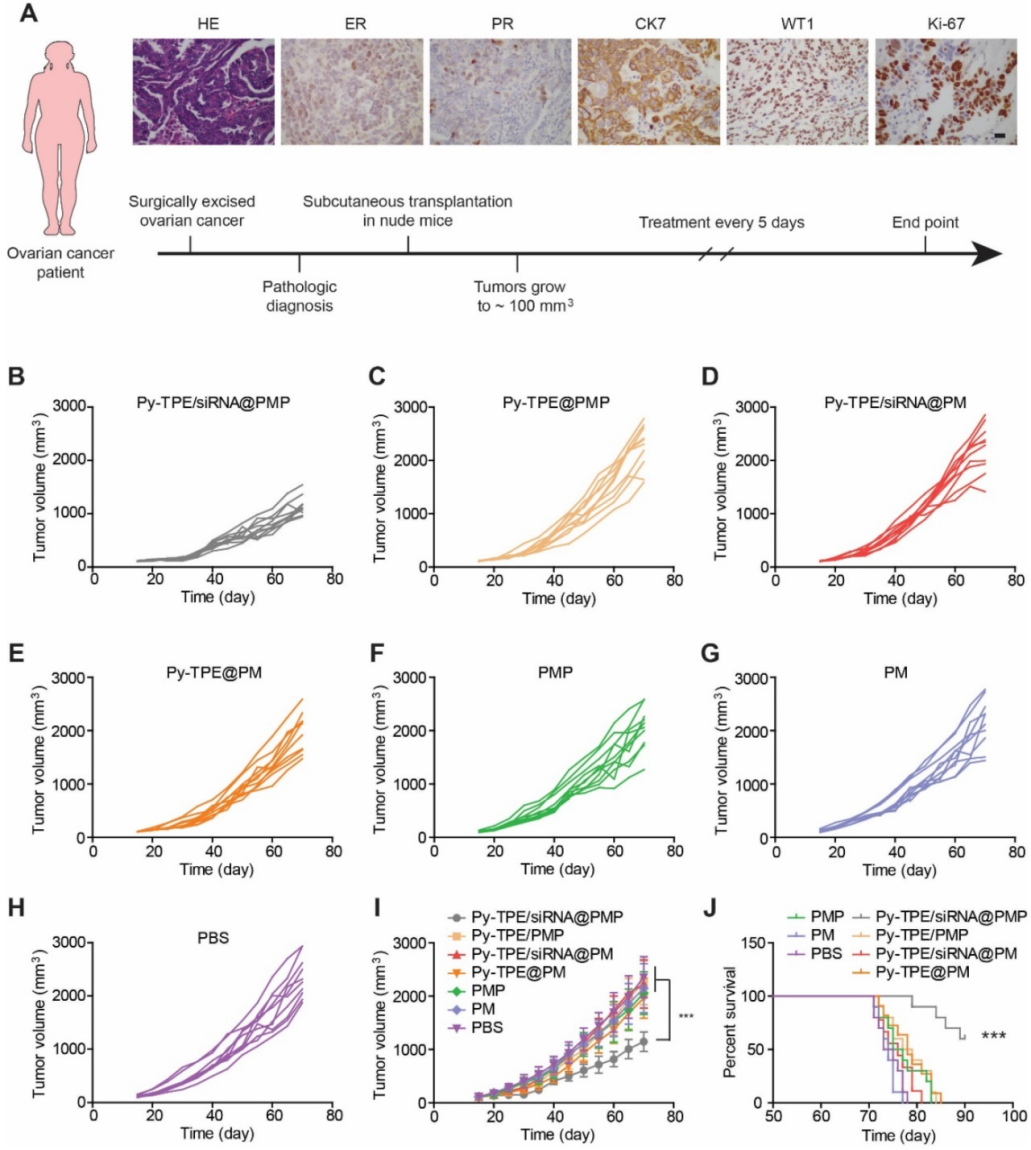
(A) The design route of PDX model and its characteristic identifications by H&E staining, estrogen receptor (ER) staining, progesterone receptor (PR) staining, immunohistochemical staining of CK7, WT1 and Ki-67. Scar bar =10 µm. The growth kinetics of PDX tumors were treated with (B) Py-TPE/siRNA@PMP, (C) Py-TPE@PMP, (D) Py-TPE/siRNA@PM, (E) Py-TPE@PM, (F) PMP, (G) PM and (H) PBS, respectively. (I) Curves of tumor volume changes during treatment. (J) Survival curves of mice receiving different treatments. *** *p* <0.001.

Establishing tumor models from patient-derived xenograft (PDX) at low passage is believed to conserve original tumor characteristics and offer the predictive insights into clinical outcomes, PDX tumor models were generated and then studied by treated with different NPs. The design route and varieties of staining of PDX models were exhibited in Figure 5A. The growth kinetics of PDX tumor showed that Py-TPE/siRNA@PMP was able to significantly inhibit tumor growth, while Py-TPE@PMP, Py-TPE/siRNA@PM, Py-TPE@PM, PMP and PM did not have such effect, which was similar to the PBS group (Figure 5B-H). In a sharp contrast, the growth kinetics of PDX tumor were significantly decreased by using Py-TPE/siRNA@PMP treatment, illustrating its striking chemotherapeutic effect on PDX models (Figure 5I). Importantly, Py-TPE/siRNA@PMP prolonged mouse survival and had the best anti-tumor effect on chemotherapy-resistant models (Figure 5J). In conclusion, the above results confirmed the synergistic combination effect of Py-TPE/siRNA@PMP on SKOV-3/PTX subcutaneous tumor model, SKOV-3/PTX intraperitoneal metastasis model and PDX tumor model and elongating the survival rate.

### The mechanism of Py-TPE/siRNA@PMP to overcome chemotherapy resistance of ovarian cancer *in vivo*

**Figure 6:**
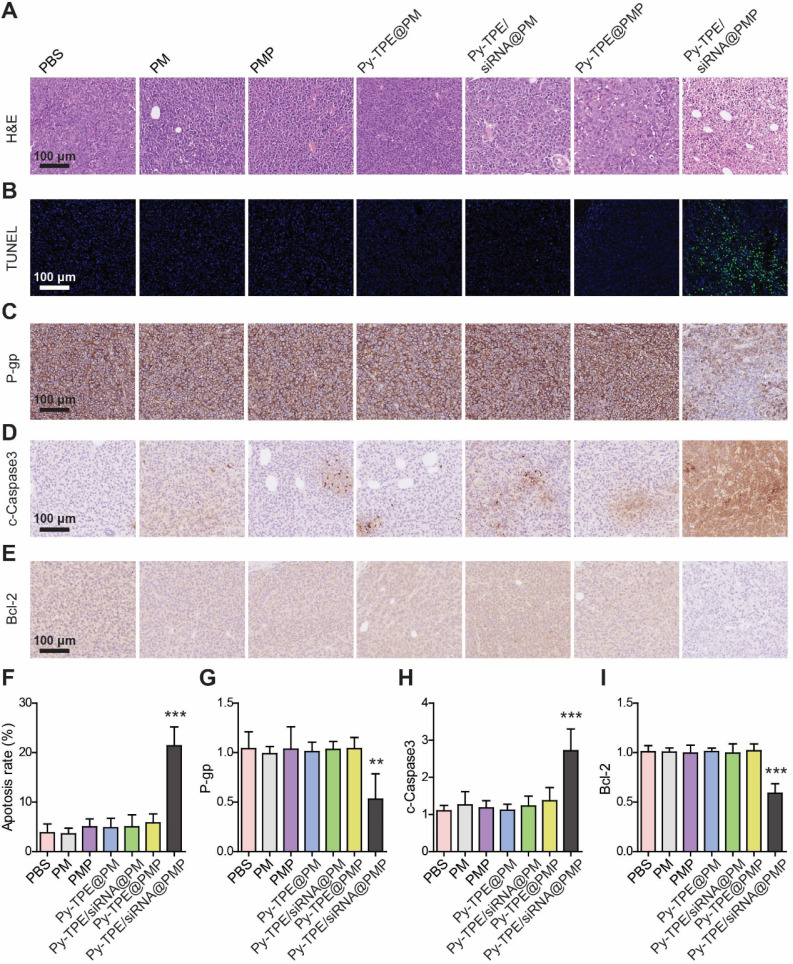
(A) H&E staining of SKOV-3/PTX tumors after treated with PM, PMP, Py-TPE@PM, Py-TPE@PMP, Py-TPE/siRNA@PM, Py-TPE/siRNA@PMP and PBS, respectively. (B) TUNEL staining of SKOV-3/PTX tumors after different treatments. The expression of (C) P-gp, (D) c-Caspase3 and (E) Bcl-2 in SKOV3/PTX tumors after different treatments. (F) Percentage of TUNEL positive cells after different treatments. Semi quantitative analysis of (G) P-gp, (H) c-Caspase3, (I) Bcl-2 in SKOV3/PTX tumors after different treatments. ** *p* <0.01, *** *p* <0.001.

To understand the therapeutic process of Py-TPE/siRNA@PMP, the morphological changes during SKOV-3/PTX cell apoptosis were investigated by using the H&E staining. As shown in Figure 6A, a large number of necrotic cells were presenting when treated with Py-TPE/siRNA@PMP group, while only a few necrotic cells existing under treatment by other groups, which indicated that either PTX or siRNA was unable to induce SKOV-3/PTX cell death (Figure 6A). Then the tumor tissues were stained with TUNEL (Figure 6B) and the percentage of TUNEL positive cells in Py-TPE/siRNA@PMP group was significantly higher than that of other groups (*p* < 0.001) (Figure 6F). Obviously, Py-TPE/siRNA@PMP finally resulted in the highest ratio of apoptosis tumor cells. Next, the P-gp expression of SKOV-3/PTX cell was evaluated and strongly expressed in Py-TPE@PMP, Py-TPE/siRNA@PM, Py-TPE@PM, PMP, PM and PBS groups (Figure 6C). However, the expression of P-gp in Py-TPE/siRNA@PMP group decreased significantly compared with other groups (*p* <0.01) (Figure 6G). It was noteworthy that Py-TPE/siRNA@PM did not bring any change to the P-gp expression (Figure 6C and 6G). We believed that high loading of Py-TPE and siRNA was achieved by encasing in the PMP to generate NPs. But without PTX, the encapsulation would be inefficient completed, leading to a reduced amount of siRNA and resulted in stronger P-gp expression. In addition, we found that the distributions of Py-TPE/siRNA@PMP and Py-TPE/siRNA@PM were different *in vivo*. Py-TPE/siRNA@PMP was mainly concentrated in tumor, while Py-TPE/siRNA@PM was more concentrated in kidney, which might explain the reason of high expression of P-gp with Py-TPE/siRNA@PM (Figure S19). In order to explore the pathway of tumor cell apoptosis, we used IHC staining to detect the expression of cleaved Caspase-3 (c-Caspase-3) (Figure 6D) and Bcl-2 (Figure 6E) in tumors. The expression of c-Caspase-3 in Py-TPE/siRNA@PMP group was the most efficient (Figure 6H), indicating a role of Py-TPE/siRNA@PMP to promote apoptosis by activating Caspase-3 pathway 47. On the contrary, the expression of Bcl-2 in Py-TPE/siRNA@PMP group was less abundant than other groups (Figure 6I), implying that Py-TPE/siRNA@PMP down-regulated Bcl-2 and promoted apoptosis 48. Hence, Py-TPE/siRNA@PMP down-regulated the expression of P-gp, and then promoted the apoptosis of chemo-resistant ovarian cancer cells through Caspase3 and Bcl-2 pathways.

### Py-TPE/siRNA@PMP reduced the side effects of chemotherapy

**Figure 7:**
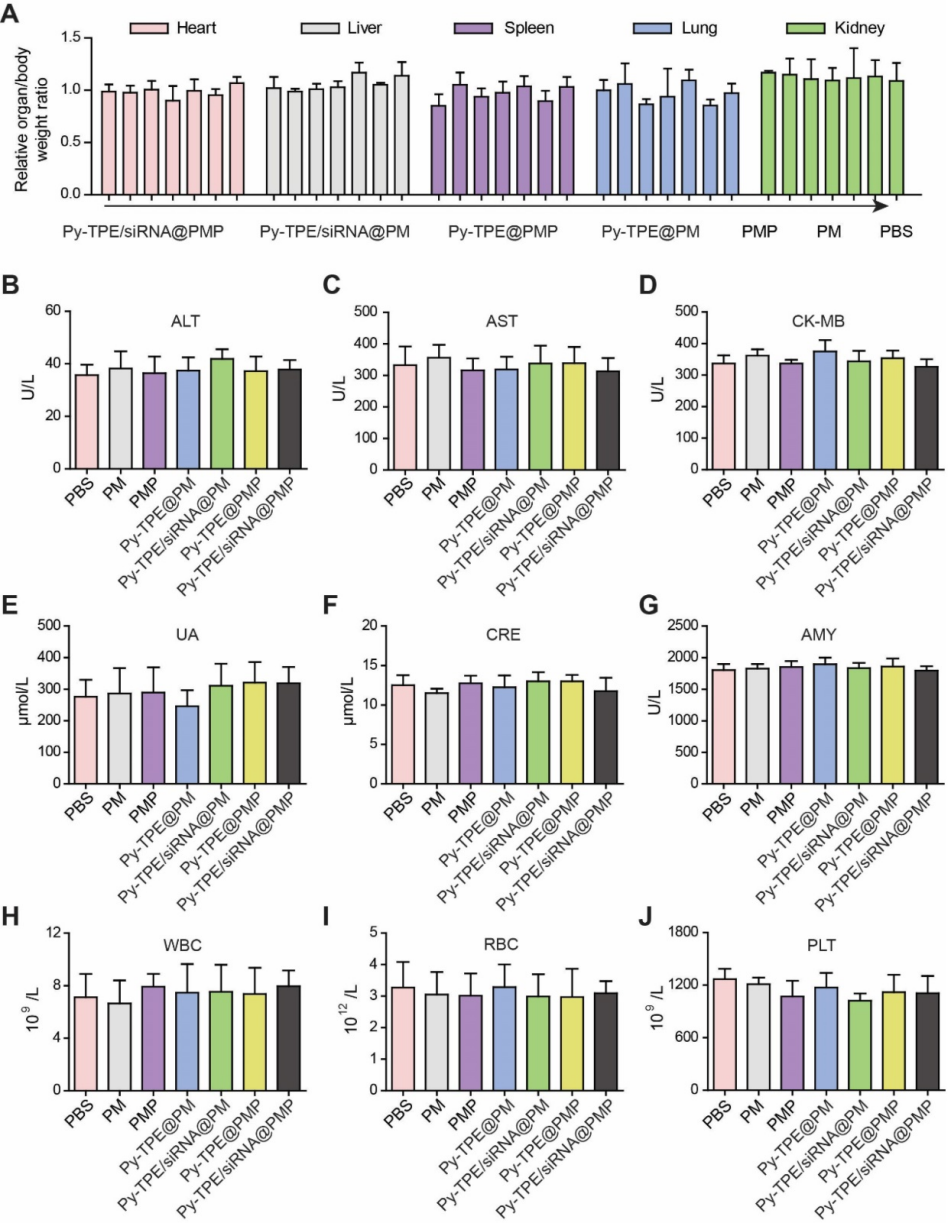
(A) Related organ/body weight ratios of SKOV-3/PTX subcutaneous tumor bearing mice treated with Py-TPE/siRNA@PMP, Py-TPE@PMP, Py-TPE/siRNA@PM, Py-TPE@PM, PMP, PM and PBS, respectively. The levels of (B) ALT, (C) AST, (D) CK-MB, (E) UA, (F) CRE and (G) AMY were measured in SKOV3/PTX tumor bearing mice treated with PBS, PM, PMP, Py-TPE@PM, Py-TPE@PMP, Py-TPE/siRNA@PM and Py-TPE/siRNA@PMP, respectively. The expressions of (H) WBC, (I) RBC, and (J) PLT in peripheral blood of SKOV3/PTX tumor bearing mice were determined after treated with PBS, PM, PMP, Py-TPE@PM, Py-TPE@PMP, Py-TPE/siRNA@PM and Py-TPE/siRNA@PMP, respectively.

The high concentration of GSH in tumor cells induced a cleavage of disulfide bond in PMP, which broke the ester bond to generate active PTX. Nevertheless, limited GSH in normal tissues or organs only produced PTX in a low concentration to prevent the corresponding side effects. In SKOV3/PTX subcutaneous tumor model, the assays of related organ/body weight ratio were consistent during the chemotherapy, without being interfered by treatment of Py-TPE/siRNA@PMP (Figure 7A), which confirmed that NPs owned good biocompatibility and mild side effect.

Both levels of alanine aminotransferase (ALT) and aspartate aminotransferase (AST) further validated that Py-TPE/siRNA@PMP was not involved in damaging the normal liver function (Figure 7B and 7C). Together with unchanged levels of CK-MB (creatine kinase-MB, heart, Figure 7D), UA and CRE (urine acid and creatinine, kidney, Figure 7E and 7F) as well as AMY (amylase, pancreas, Figure 7G), the results verified low side effect of Py-TPE/siRNA@PMP on different organs and rendered a reliable prodrug to treat the fatal ovarian cancer *in vivo*. On the other hand, different blood cells including white blood cells (WBC) (Figure 7H), red blood cells (RBC) (Figure 7I), and platelets (PLT) (Figure 7J), were monitored to investigate the effect of Py-TPE/siRNA@PMP on their functional performance. It was found that there was almost no reduction of all bloody cells amount in the treatment of SKOV-3/PTX tumor models, indicating the low side effect of Py-TPE/siRNA@PMP.

## Conclusion

Establishing a reliable chemotherapy strategy for maintaining of the tumor drug sensitivity and reducing side effects in pursuit of improved success rate of ovarian cancer treatment remains a challenge. In this vein, we constructed a complex structure of Py-TPE and P-gp siRNA, encapsulated them with PMP and finally obtained Py-TPE/siRNA@PMP NPs by self-assembly. The Py-TPE/siRNA@PMP was delivered into the cell with the help of macropinocytosis and lit up the ovarian cancer cells with high fluorescence contrast and resolution. The therapeutic study in SKOV-3/PTX cells demonstrated a synergistic effect of enhanced PTX treatment upon gene regulation by P-gp siRNA. In SKOV-3/PTX subcutaneous, intraperitoneal metastasis and PDX tumor models, Py-TPE/siRNA@PMP specifically accumulated in the tumor sites by EPR effect after intravenous injection, and significantly inhibited the ovarian cancer growth by overcoming the chemotherapy resistance. IHC staining further validated that Py-TPE/siRNA@PMP promoted cellular apoptosis through regulation of Casepas-3 and Bcl-2. Moreover, the organ/body weight ratios and blood cells indexes proved the limited side effect of Py-TPE/siRNA@PMP to the normal organs during chemotherapy. These results indicated promising NPs for tumor imaging and gene-regulated chemotherapy for ovarian cancer. In conclusion, it could be envisaged that the design of Py-TPE/siRNA@PMP would provide guidance for the future development of therapeutic reagents on the basis of combinational polymeric prodrug and AIEgens for efficient chemotherapy in drug-resistant cancer with the minimal side effects.

## Material and methods

### Synthesis of PMP and Py-TPE

PTX-SS-N_3_, PEG-b-PMPMC (PM), and PEG-b-PMPMC-g-PTX (PMP) were synthesized according to our previous literature 31. Py-TPE was synthesized following the procedures in the literatures 33.^ 1^H NMR spectra were measured on a Bruker ARX 400 NMR spectrometer with chlroform-d (CDCl_3_-d) as the solvent and tetramethylsilane (TMS) as the internal reference. High resolution mass spectra (HRMS) were recorded on a Bruker microTOF II mass spectrometer system operating in MALDI-TOF mode.

### Preparation of Py-TPE/siRNA@PMP NPs

PMP and Py-TPE were dissolved in DMF at concentrations of 10 mg/mL and 2.5 mg/mL, respectively. Subsequently, Py-TPE (800 μg in 320 μL) and siRNA (16.672 μg in 80μL) dissolved in aqueous solution were mixed in a weight ratio of 48:1 and then lightly stirred for 6 h. PMP (1 mg in 100 μL) was added into the mixed solution and stirred for 12 h. Next, 1 mL of ultrapure water was gradually added to the solution, and the mixture was also stirred for 12 h. Finally, this solution was transferred into a dialysis tube (cutoff Mw 1000) and dialyzed against ultrapure water for 24 h. The ultrapure water was refreshed every 4 h to remove the DMF.

### Electrophoresis

The nucleic acid (P-gp siRNA) loading of NPs was detected by agarose gel electrophoresis. Briefly, siRNA (5.21 μg in 80 μL) complexed with different amounts of Py-TPE in varying weight ratios (from 12 to 96) was incubated with DMF for 30 min. Then, PMP was added to the mixed solution. For siRNA samples in DMF, electrophoresis was conducted without extracting siRNA from DMF into aqueous solution. The loading dye (Invitrogen) was added into the moderate samples and electrophoresed on a 2% agarose gel at 100 V for 1 h. Ultraviolet was used to image the gel and the bands were analyzed.

### Characterization of NPs

UV-Vis: An Agilent Cary 60 UV/Visible Spectrometer was used to measure the UV-Vis absorption spectra. Fluorescence measurement: The fluorescence of NPs was measured by an Agilent Cary Eclipse Fluorescence Spectrophotometer. Particle size measurement: the particle size of NPs was determined by Nano-ZS ZEN3690 (Malvern Instruments) in PBS buffer at 25 °C. Transmission electron microscope (TEM) images: TEM was imaged by a FEI Tecnai G2 12 TEM instrument at an accelerating voltage of 100 kV. 1% (w/v) phosphotungstic acid solution was used to stain the samples. Zeta measurement: The Zeta potential of NPs was measured by Nano-ZS ZEN3690 (Malvern Instruments) at 25 °C.

### The drug loading capacity of Py-TPE and PTX in NPs

1 mL of NPs was lyophilized and dissolved in DMF to measure the drug loading capacity of Py-TPE in Py-TPE/siRNA@PMP NPs. After stirring for 1 h, the loading capacity of Py-TPE in Py-TPE/siRNA@PMP was tested using UV-Vis. The drug loading capacity was calculated as follows: drug loading capacity = (weight of loaded drug/total weight of polymer and loaded drug) ×100%. The drug loading capacity of PTX in Py-TPE/siRNA@PMP NPs was analyzed according to our previous literatures 31.

### *In vitro* cell imaging

SKOV-3 cells were cultured in DMEM medium containing 1% antibiotics (penicillin-streptomycin, 10000 U/mL) and 10% fetal bovine serum at 37 °C under 5% CO_2_.SKOV-3 cells were incubated with NPs in an atmosphere of 5% CO_2_ at 37 °C. After incubating for 4 hours, the culture medium was removed, and the cells were washed three times with PBS. Then, the cells were fixed with 4% paraformaldehyde and the fluorescence of NPs was examined (Py-TPE: Ex =405 nm, Em =600 nm).

### CCK-8 assay

CCK-8 (Cell Counting Kit-8) was employed for cytotoxicity assessment of SKOV-3 cells. 1 × 10^4^ of cell suspension was seeded into each well of a 96-well plate and incubated for 24 h. Various concentrations of NPs were added to the cells and incubated for 24 h. The medium was removed and fresh medium (100 μL) was added to the cells. Then 10 μL of CCK-8 solution was added to each well and incubated for another 1 h. The UV-Vis absorption was measured at 450 nm.

### Western blot analysis

After separation and membrane transfer, the protein sample was incubated with the primary antibody. The primary antibody (P-gp, ab196500, Abacm, 1:500 dilution; β-actin, KM9001, SUNGENE BIOTECH, 1:10000 dilution) was incubated overnight at 4 °C. After incubation with HRP-conjugated secondary antibody, immunoreactivity was detected using ECL substrates and recorded through the ChemiDoc imaging system (Bio-Rad, Hercules, USA).

### Animals

The experimental animal scheme strictly complied with the requirements of the experimental animal ethics committee of Tongji hospital, Tongji Medical College, Huazhong University of Science and Technology, and conformed to the principles of animal protection, animal welfare and ethics. BALB/c nude mice were purchased from HFK Bioscience Co. (Beijing, China) and fed normally with unlimited water and food.

### Subcutaneous tumor model

Briefly, 1 ×10^6^ SKOV-3 cells were subcutaneously inoculated into the right anterior side of female BALB/c nude mice. Tumor growth was measured using a vernier caliper. The tumor volume was calculated as follows: volume = 0.5 × (tumor length) × (tumor width)^2^. When the volume of SKOV-3 tumor reached around 50 mm^3^, SKOV-3 tumor-bearing mice were randomly divided into 7 groups. Mice were injected with Py-TPE/siRNA@PMP (300 μg), Py-TPE@PMP (295 μg), siRNA/Py-TPE@PM (220 μg), Py-TPE@PM (215 μg), PMP (235 μg), PM (160 μg) and PBS (200 μL) *via* the tail vein, respectively. All mice were treated every other day, and the growth kinetics and weight changes of tumor were recorded.

### Intraperitoneal metastasis model

After intraperitoneal injection of 10^6^ EGFP-SKOV-3 cells, 8-week-old nude mice were randomly divided into 7 groups: Py-TPE/siRNA@PMP, Py-TPE@PMP, siRNA/Py-TPE@PM, Py-TPE@PM, PMP, PM and PBS. The intraperitoneal drug injection was started on the third day of EGFP-SKOV-3 cell transplantation and carried out every two days. After 6 cycles of treatment, the abdominal cavity of mouse was opened, and the blood ascites and metastasis were recorded and observed. EGFP-SKOV-3 cells were able to express green fluorescent protein, which can be traced and quantified by IVIS Spectrum (PerkinElmer, EGFP: Ex=500 nm, Em =540 nm).

### PDX tumor model

The tumor tissue of ovarian cancer was cut into 1mm^3^ tissue blocks and transplanted subcutaneously in nude mice. When the tumor grew to about 100 mm^3^, it was randomly divided into 7 groups: Py-TPE/siRNA@PMP, Py-TPE@PMP, siRNA/Py-TPE@PM, Py-TPE@PM, PMP, PM and PBS. Treatment was given every 5 days until the end of the experiment. Tumor volume and mouse survival were recorded.

### *In vivo* tumor imaging and bio-distribution

When the tumor grew to 500 mm^3^, SKOV-3 tumor-bearing mice were injected with 300 μg of Py-TPE/siRNA@PMP *via* tail vein. The mice were imaged by the IVIS Spectrum (Py-TPE: Ex =405 nm, Em =600 nm) at the time points of 3 h, 6 h, 12 h, 24 h and 48 h after injection and then sacrificed for tissue distribution study respectively. The tumors and major organs (heart, liver, spleen, lungs, and kidneys) of each mouse were collected and imaged.

### Statistical analysis

Statistical analysis was also carried out using GraphPad Prism 5.0 software to perform two-sided Student's t-test. All results were presented as mean ±SD as indicated. Statistical differences were defined as *n.s.* not significant, * *p*<0.05, ** *p*<0.01 and *** *p*<0.001.

## Supplementary Material

Supplementary figures.

## Abbreviations

UV-Vis: ultraviolet-visible
PTX: paclitaxel
NPs: nanoparticles
AIEgen: aggregation-induced emission fluorogen
NDDS: nanoparticle drug delivery system
EPR: enhanced permeability and retention
GSH: glutathione
PMP: poly (ethylene glycol)-b-poly(5-mthyl-5-propargyl-1,3-dioxan-2-one)-g-paclitaxel
P-gp: P-glycoprotein
PBS: phosphate-buffered saline
TEM: transmission electron microscopy
DLS: dynamic light scattering
CLSM: confocal laser scanning microscope
SKOV-3/PTX cells: PTX resistance SKOV-3 cells
PDX: patient-derived xenografts
CPZ: clathrin-mediated endocytosis inhibitor
Filipin: caveolae-mediated endocytosis inhibitor
EIPA: macropinocytosis inhibitor
CMFDA: 5-chloromethylfluorescein diacetate
AST: aspartate
ALT: alanine aminotransferase
GLU: glucose
CRE: creatine
WBC: white blood cells
RBC: red blood cell
PLT: platelet
CK-MB: creatine kinase-MB
UA: urine acid
CRE: creatinine
AMY: andamylase
