# Supplementary material for: Biocompatible AIEgen/p-glycoprotein siRNA@reduction-sensitive paclitaxel polymeric prodrug nanoparticles for overcoming chemotherapy resistance in ovarian cancer
Source: Theranostics. 2021 Jan 27;11(8):3710–24. doi: 10.7150/thno.53828 (PMC7914360; doi:10.7150/thno.53828)
Supplement: Supplementary file 1 — Supplementary figures. [file thnov11p3710s1.pdf]

Biocompatible AIEgen/p-glycoprotein siRNA@reduction-sensitive paclitaxel polymeric prodrug nanoparticles for overcoming chemotherapy resistance in ovarian cancer

Jun Wu[1*], Quan Wang[1*], Xiaoqi Dong[1], Min Xu[1], Juliang Yang[1], Xiaoqing Yi[3], Biao Chen[2], Xiyuan Dong[2], Ying Wang[4], Xiaoding Lou [✉1], Fan Xia[1], Shixuan Wang[✉2] and Jun Dai[✉2]

[1] Engineering Research Center of Nano-Geomaterials of the Ministry of Education, Faculty of Materials Science and Chemistry, China University of Geosciences, Wuhan 430074, China.

[2] Department of Obstetrics and Gynecology, Tongji Hospital, Tongji Medical College, Huazhong University of Science and Technology, Wuhan 430030, China.

[3] College of Pharmacy, Gannan Medical University, Ganzhou 341000, China

[4] Department of Pathology, Tongji Hospital, Tongji Medical College, Huazhong University of Science and Technology, Wuhan 430030, China.

* These authors contributed equally to this work.

✉ Corresponding author: louxiaoding@cug.edu.cn (Xiaoding Lou); shixuanwang@tjh.tjmu.edu.cn (Shixuan Wang); jundai@tjh.tjmu.edu.cn (Jun Dai)

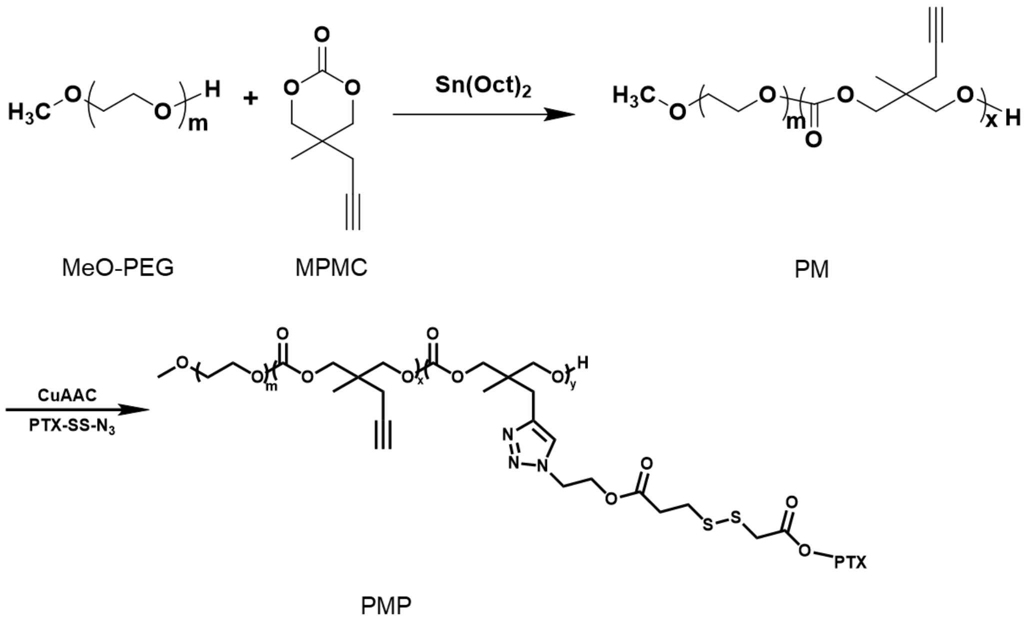

Scheme S1. The sythetic route of PMP.

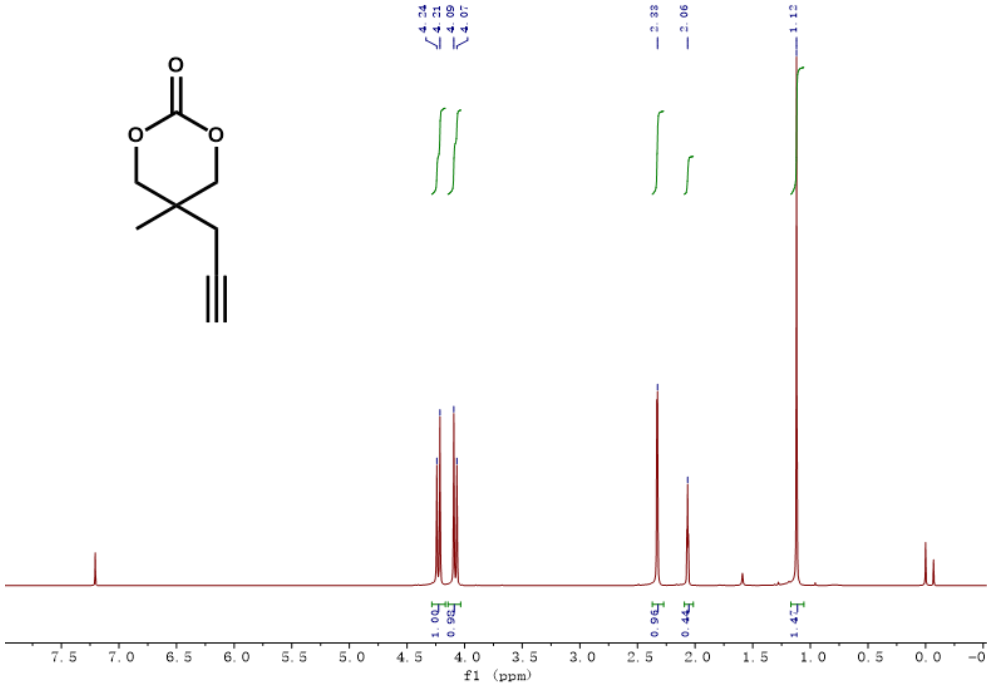

Figure S1. ^{1}H NMR spectra of MPMC.

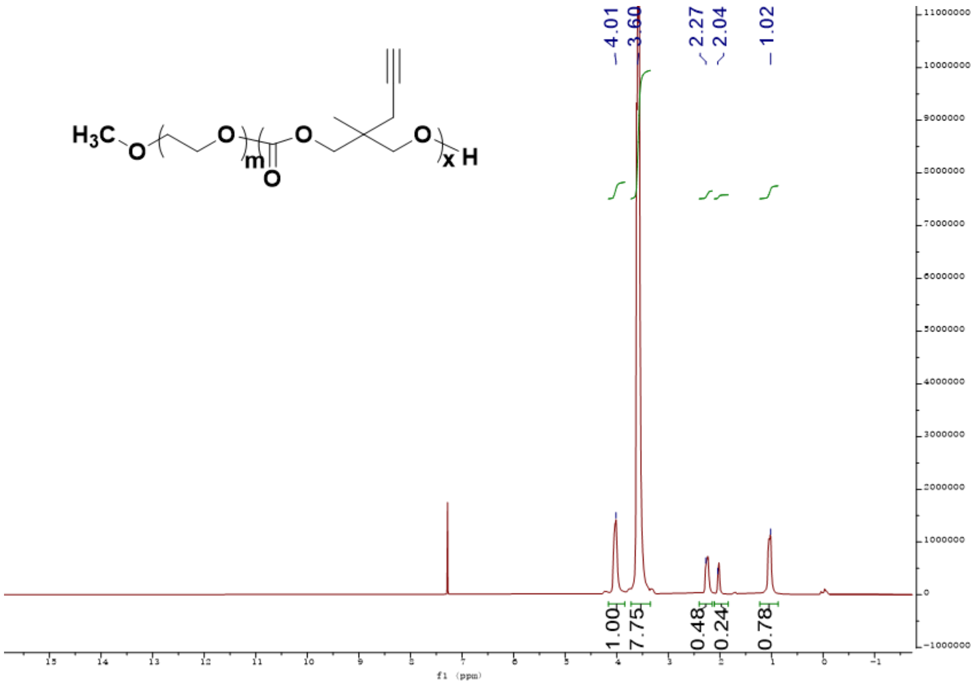

Figure S2. ^{1}H NMR spectra of PM.

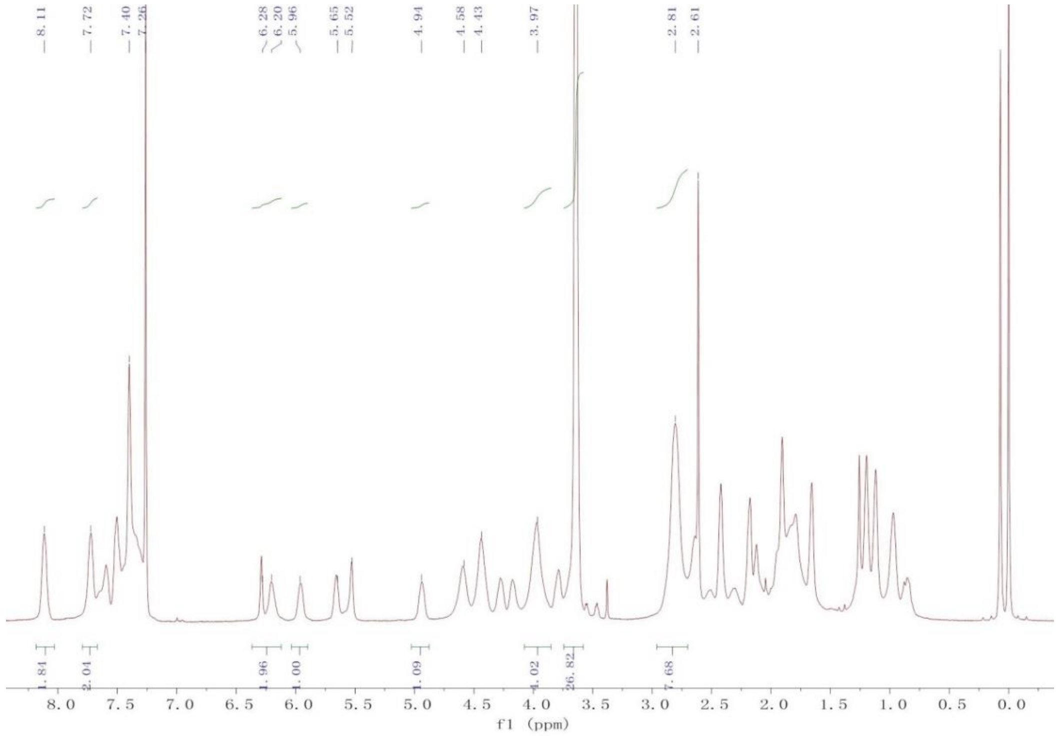

Figure S3. ^{1}H NMR spectra of PMP.

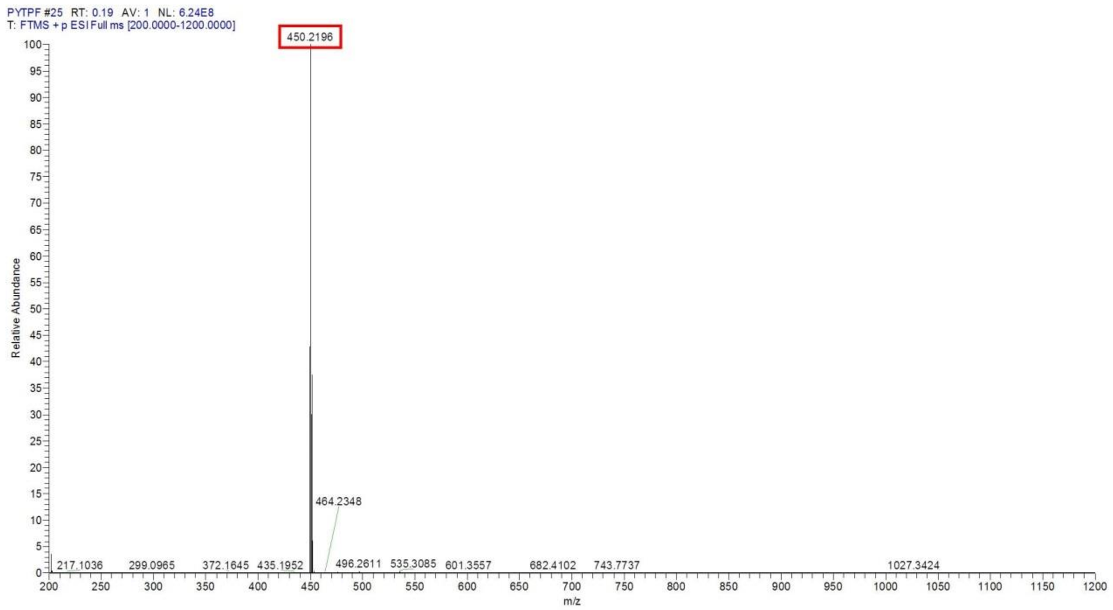

Figure S4. Mass spectrometry of Py-TPE.

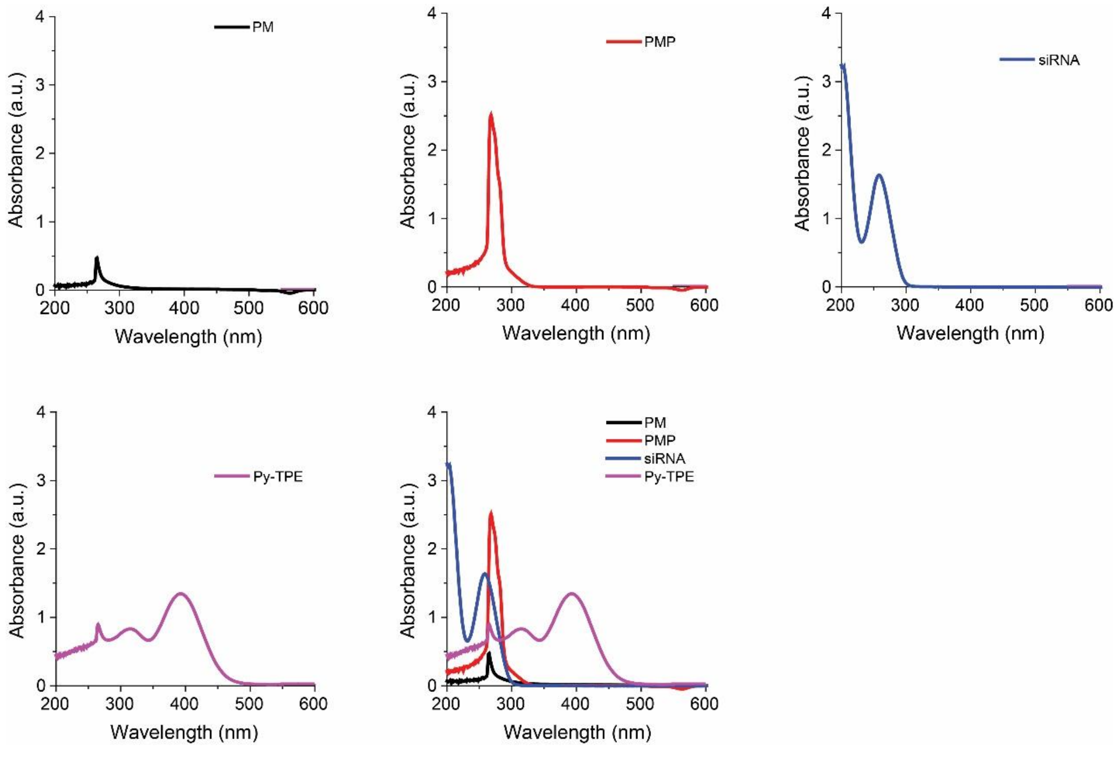

Figure S5. UV-vis characteristic absorption spectra of PM, PMP, P-pg siRNA and Py-TPE.

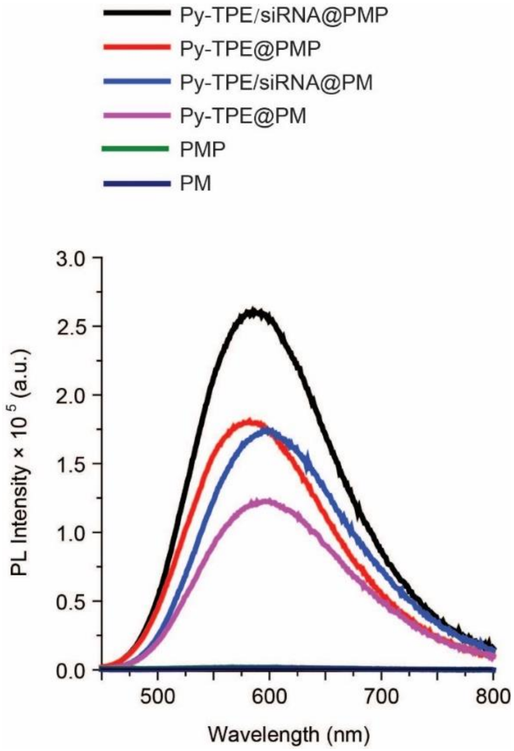

Figure S6. Fluorescence spectrum of Py-TPE/siRNA@PMP, Py-TPE@PMP, Py-TPE/siRNA@PM, Py-TPE@PM, PMP and PM.

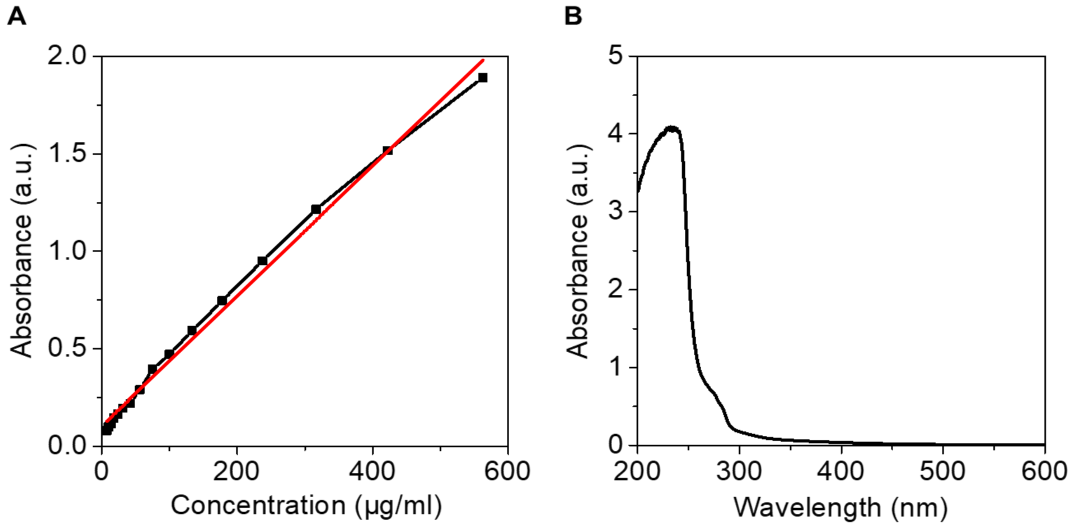

Figure S7. (A) A linear response range between PTX concentration and UV-vis absorbance. The corresponding equation is A= 0.00334C+ 0.1026, where A is the UV-vis absorbance and C is the concentration of PTX. (B) The UV-vis absorbance of 0.5 ml PMP.

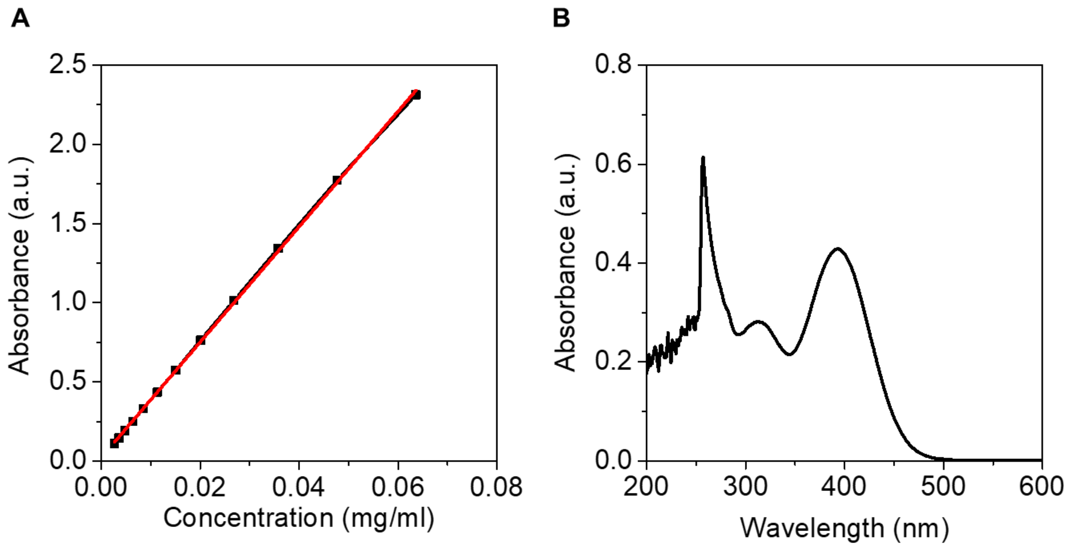

Figure S8. (A) A linear response range between PyTPE concentration and UV-vis absorbance. The corresponding equation is A= 36.422C+ 0.0225, where A is the UV-vis absorbance and C is the concentration of PyTPE. (B) The UV-vis absorbance of 0.05 ml PMP.

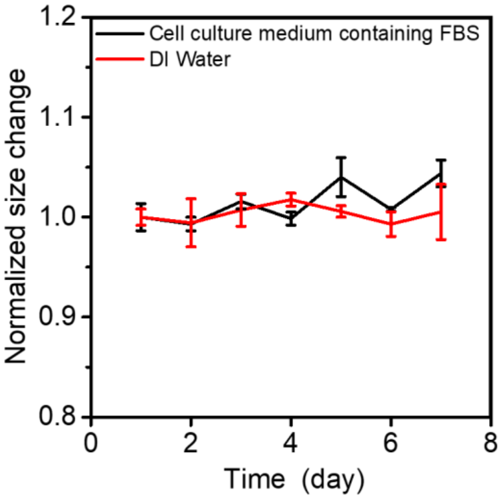

Figure S9. Normalized size change of Py-TPE/siRNA@PMP in DI water and cell culture medium containing FBS for 0-7 days.

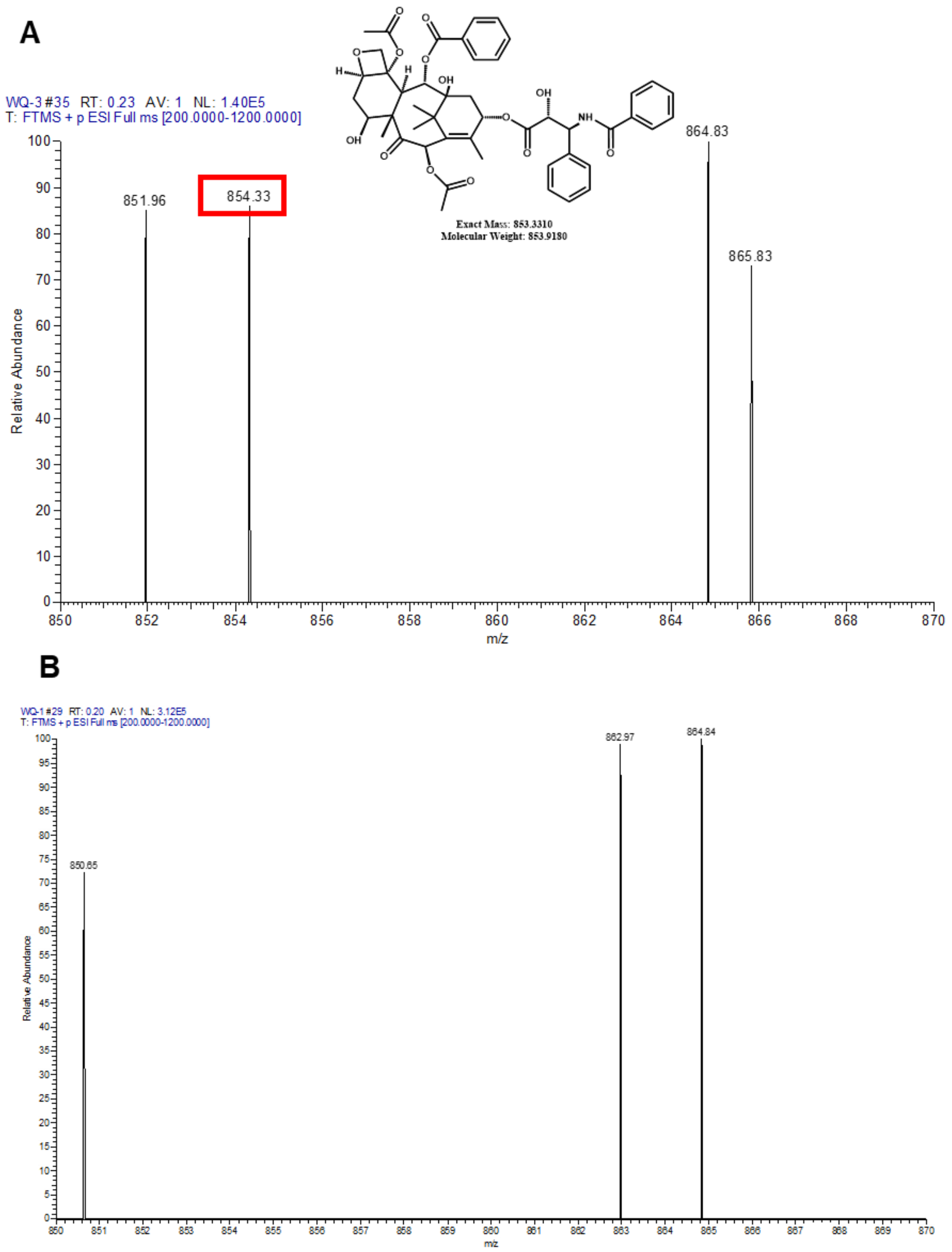

Figure S10. Mass spectroscopy of PTX releasing with GSH concentrations of (A) 10 mM and (B) 10 µM.

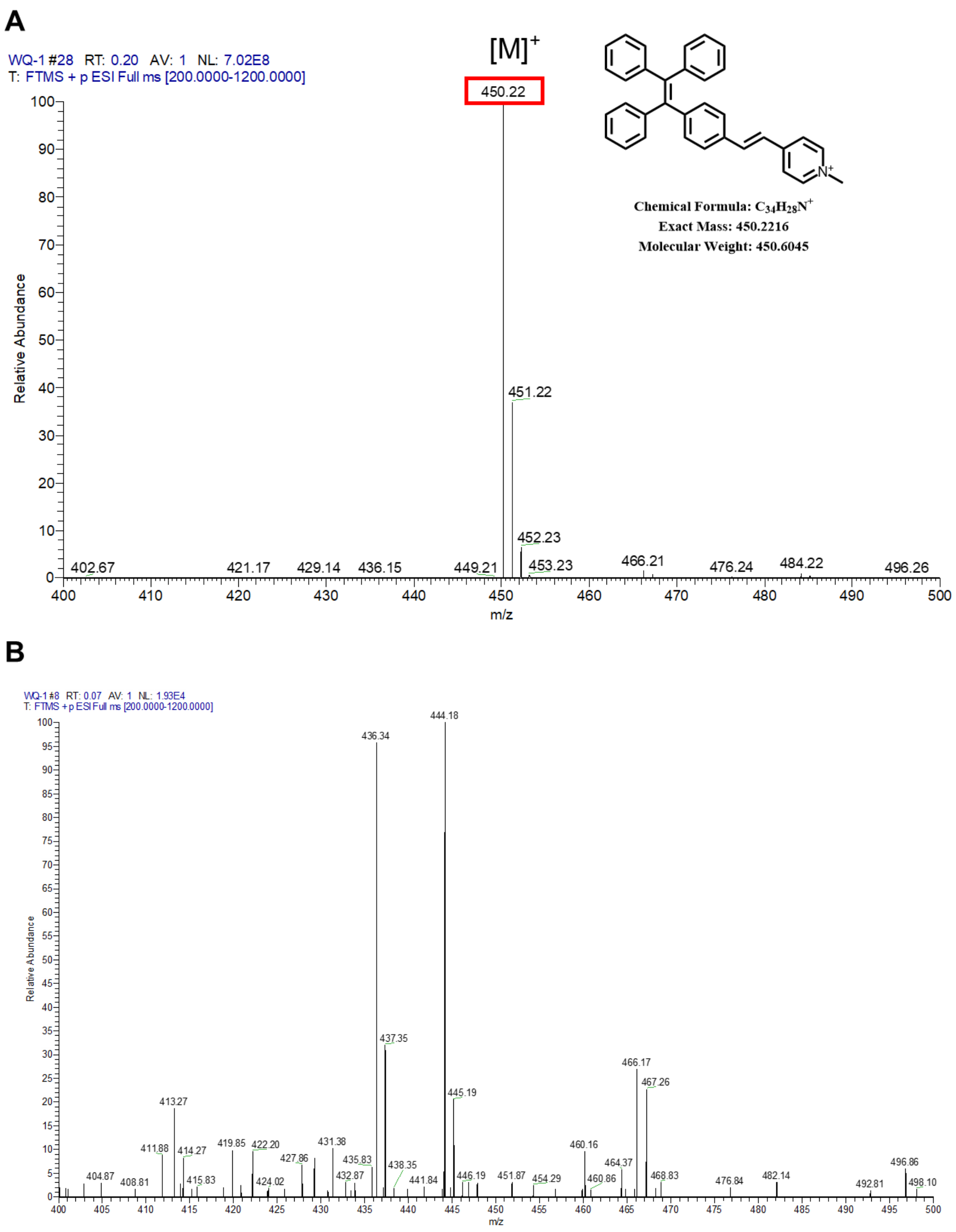

Figure S11. Mass spectroscopy of PyTPE releasing with GSH concentrations of (A) 10 mM and (B) 10 μM.

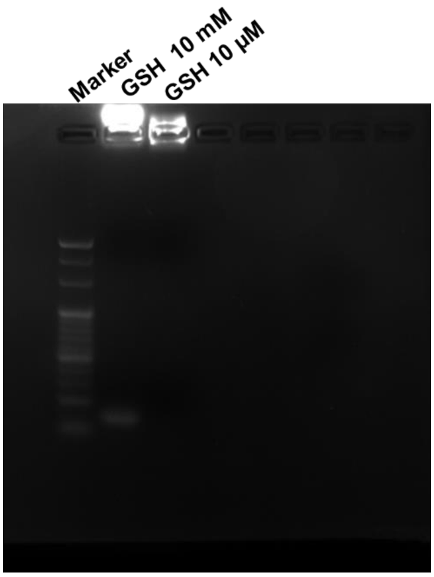

Figure S12. Agarose gel analysis of siRNA releasing with GSH concentrations of 10 mM and 10 μM.

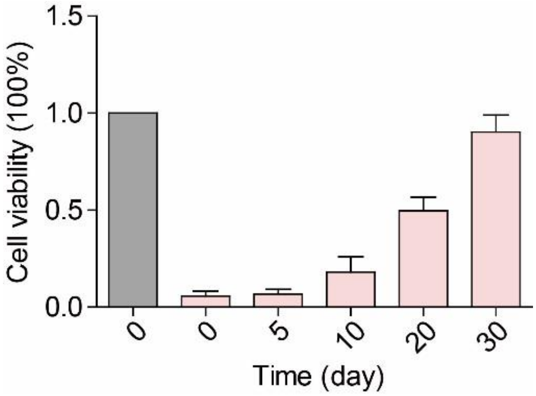

Figure S13. PTX induced resistance of SKOV-3 to chemotherapy.

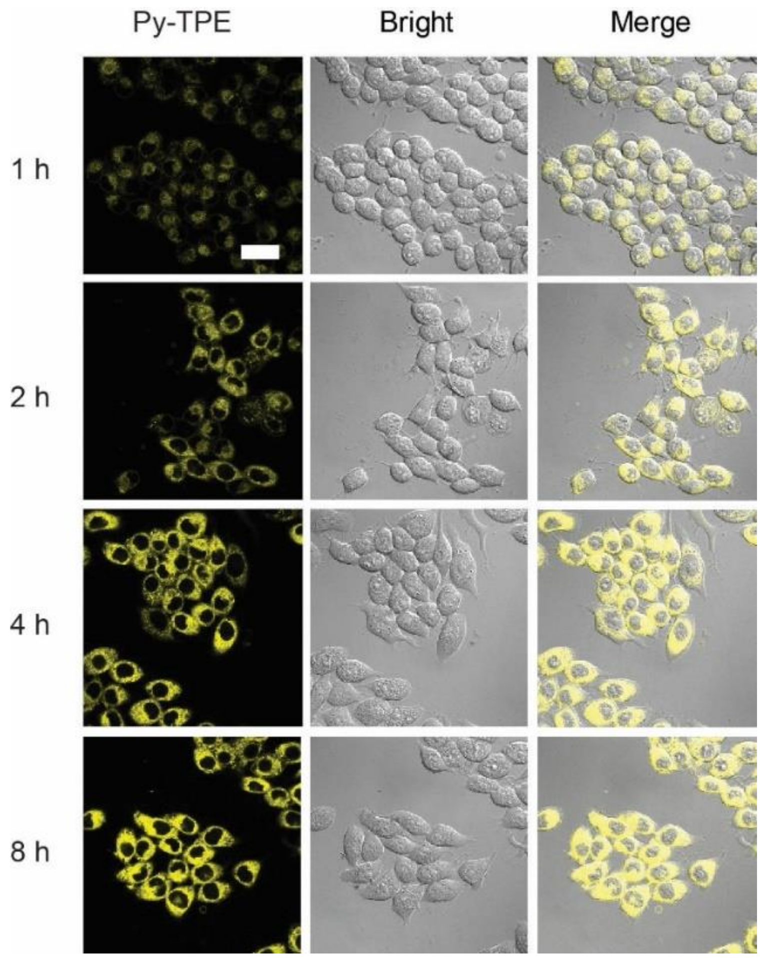

Figure S14. SKOV-3/PTX cells were cultured with Py-TPE/siRNA@PMP (30μg/mL) for 1, 2, 4 and 8 h, respectively. and then CLSM were used to detect the fluorescence signal. Ex =430 nm, Em=600 nm. Scale bar: 10 nm.

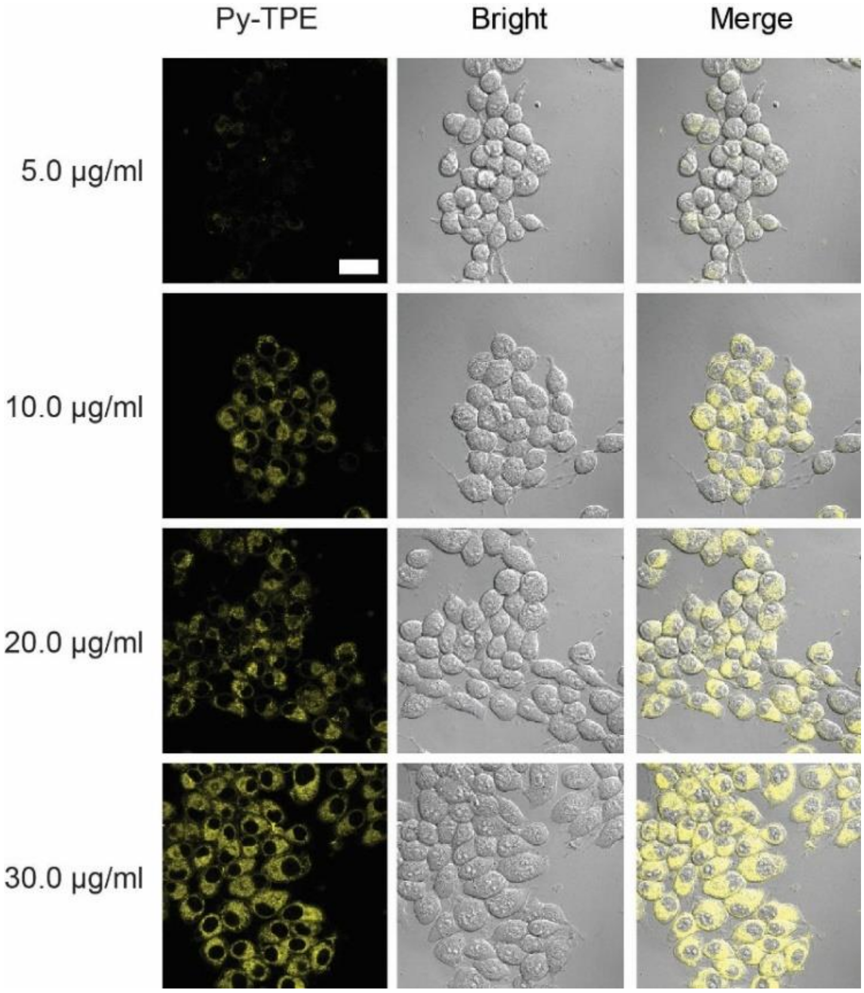

Figure S15. SKOV-3/PTX cells were cultured with different concentrations of Py-TPE/siRNA@PMP for 4 h, and then CLSM was used to detect the fluorescence signal. Scale bar: 10 nm. Py-TPE: Ex =430 nm, Em=600 nm.

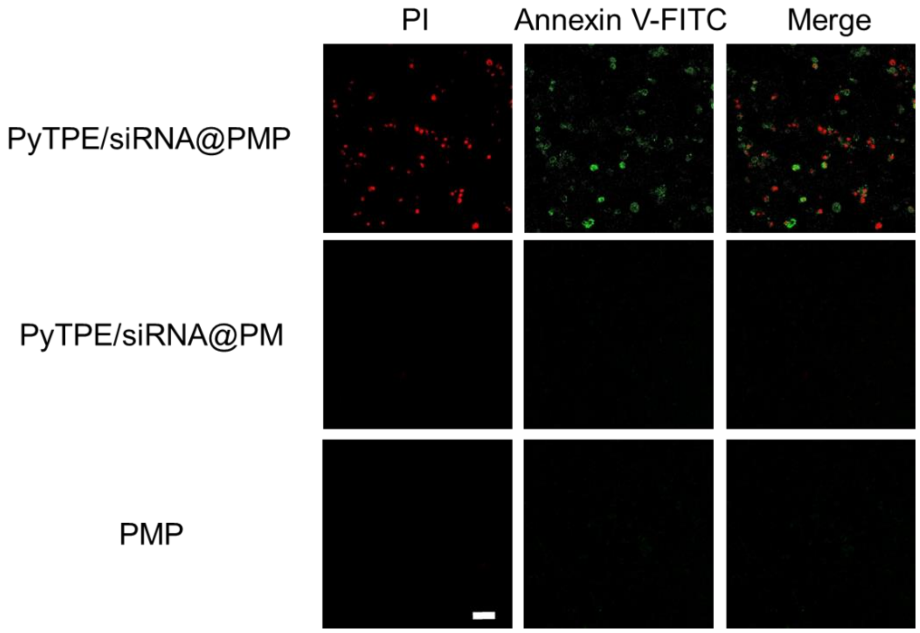

Figure S16. PI and Annexin V-FITC staining assay by using PMP, Py-TPE/siRNA@PM and Py-TPE/siRNA@PMP, respectively. Scare bar: 50 μm.

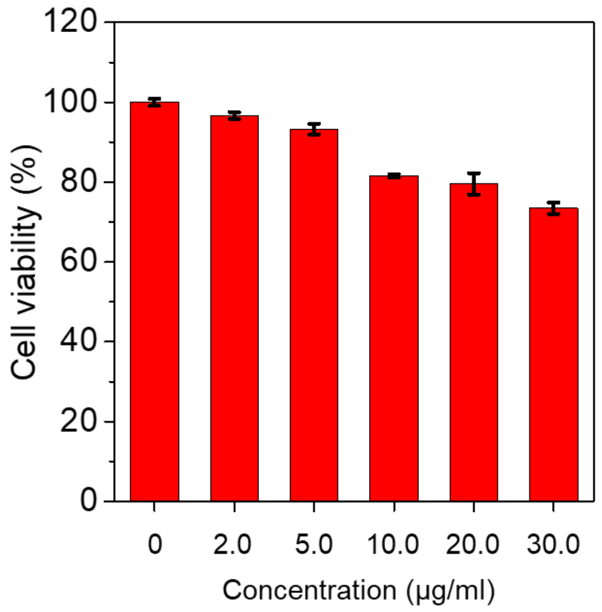

Figure S17. Cell viability of HLF cells under treatment with different concentrations of Py-TPE/siRNA@PMP NPs.

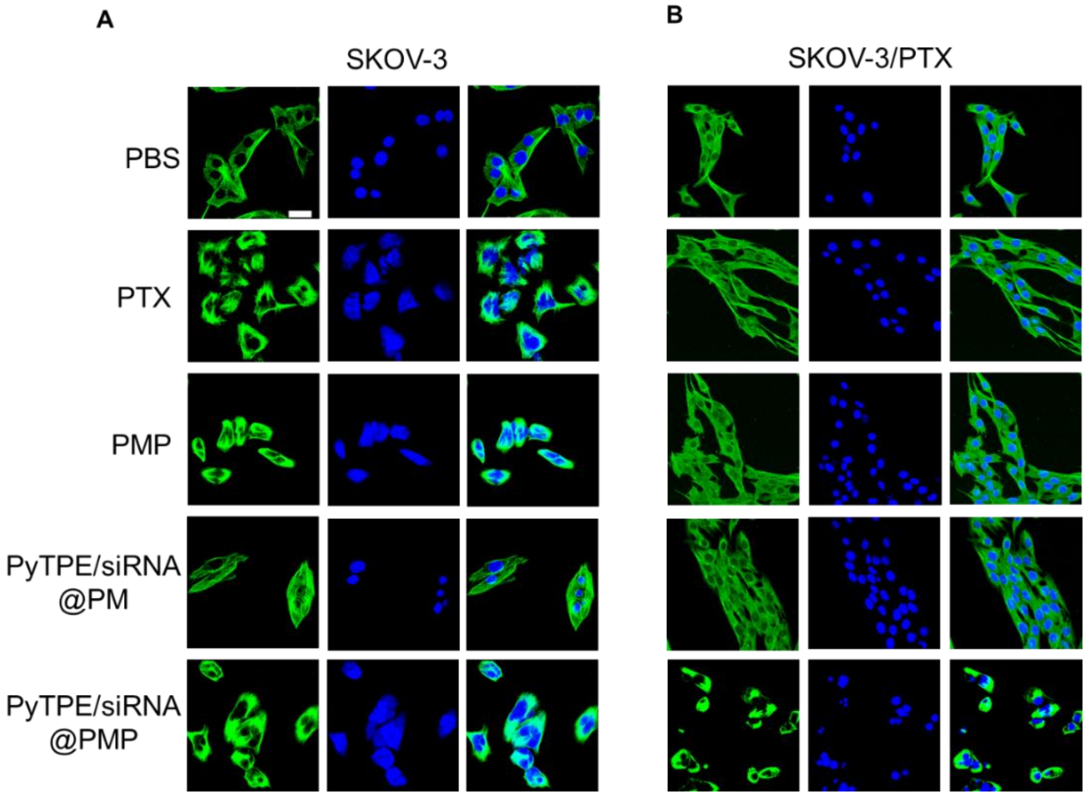

Figure S18. Microtubes in (A) SKOV-3 cells and (B) SKOV-3/PTX cells under the treatment of PBS, PTX, PMP, Py-TPE/siRNA@PM and Py-TPE/siRNA@PMP. Scale bar: 20 μm.

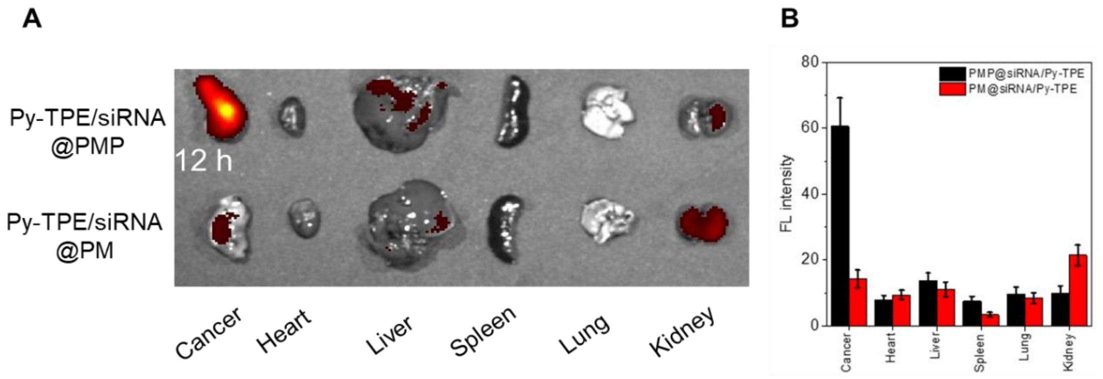

Figure S19. (A) The images of tumor and organs after Py-TPE/siRNA@PMP and Py-TPE/siRNA@PM were injected into the mice through tail vein for 12 h, respectively. (B) The relative fluorescence intensity of (A).

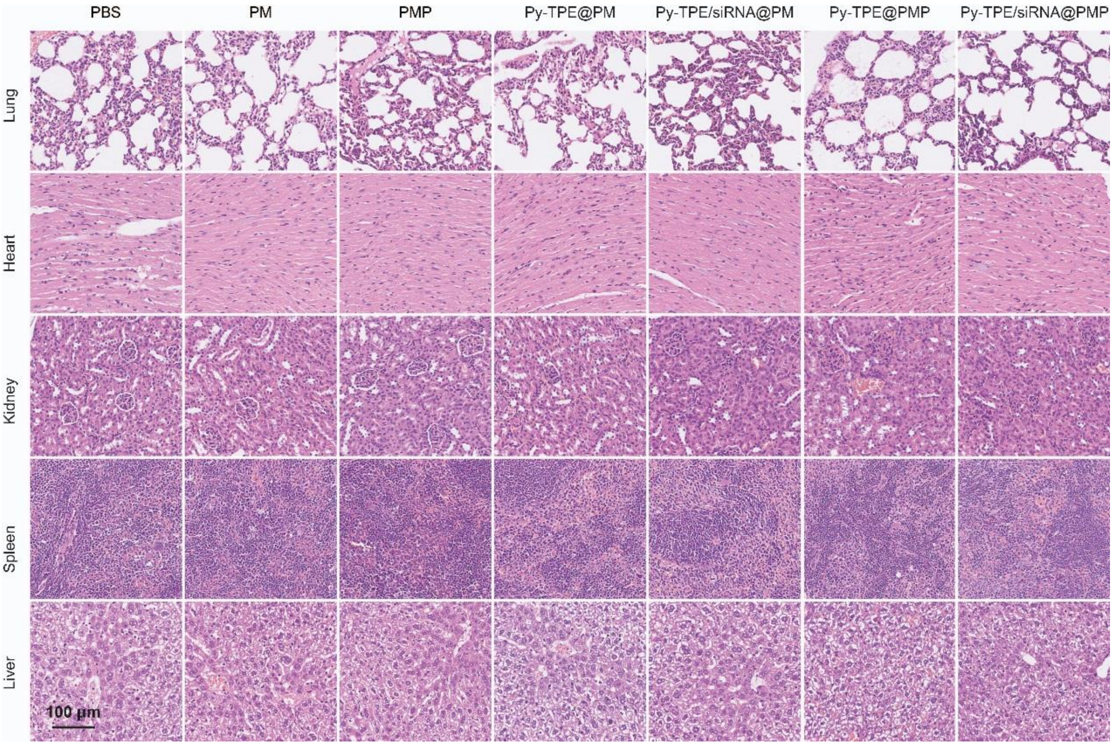

Figure S20. Histological analysis of heart, liver, spleen, lung and kidney in SKOV3/PTX tumor bearing mice after treated with PBS, PM, PMP, Py-TPE@PM, Py-TPE@PMP, Py-TPE/siRNA@PM and Py-TPE/siRNA@PMP, respectively. Scale bar: 100 nm.
